# Proteotyping bacteria: Characterization, differentiation and identification of pneumococcus and other species within the Mitis Group of the genus *Streptococcus* by tandem mass spectrometry proteomics

**DOI:** 10.1371/journal.pone.0208804

**Published:** 2018-12-10

**Authors:** Roger Karlsson, Lucia Gonzales-Siles, Margarita Gomila, Antonio Busquets, Francisco Salvà-Serra, Daniel Jaén-Luchoro, Hedvig E. Jakobsson, Anders Karlsson, Fredrik Boulund, Erik Kristiansson, Edward R. B. Moore

**Affiliations:** 1 Department of Infectious Diseases, Institute of Biomedicine, Sahlgrenska Academy of the University of Gothenburg, Gothenburg, Sweden; 2 Nanoxis Consulting AB, Gothenburg, Sweden; 3 Centre for Antibiotic Resistance Research (CARe), University of Gothenburg, Gothenburg, Sweden; 4 Microbiology, Department of Biology, University of the Balearic Islands, Palma de Mallorca, Spain; 5 Culture Collection University of Gothenburg (CCUG), Sahlgrenska Academy of the University of Gothenburg, Gothenburg, Sweden; 6 Department of Mathematical Sciences, Chalmers University of Technology, Gothenburg, Sweden; Fred Hutchinson Cancer Research Center, UNITED STATES

## Abstract

A range of methodologies may be used for analyzing bacteria, depending on the purpose and the level of resolution needed. The capability for recognition of species distinctions within the complex spectrum of bacterial diversity is necessary for progress in microbiological research. In clinical settings, accurate, rapid and cost-effective methods are essential for early and efficient treatment of infections. Characterization and identification of microorganisms, using, bottom-up proteomics, or “proteotyping”, relies on recognition of species-unique or associated peptides, by tandem mass spectrometry analyses, dependent upon an accurate and comprehensive foundation of genome sequence data, allowing for differentiation of species, at amino acid-level resolution. In this study, the high resolution and accuracy of MS/MS-based proteotyping was demonstrated, through analyses of the three phylogenetically and taxonomically most closely-related species of the Mitis Group of the genus *Streptococcus*: i.e., the pathogenic species, *Streptococcus pneumoniae* (pneumococcus), and the commensal species, *Streptococcus pseudopneumoniae* and *Streptococcus mitis*. To achieve high accuracy, a genome sequence database used for matching peptides was created and carefully curated. Here, MS-based, bottom-up proteotyping was observed and confirmed to attain the level of resolution necessary for differentiating and identifying the most-closely related bacterial species, as demonstrated by analyses of species of the *Streptococcu*s Mitis Group, even when *S*. *pneumoniae* were mixed with *S*. *pseudopneumoniae* and *S*. *mitis*, by matching and identifying more than 200 unique peptides for each species.

## Introduction

Traditional characterization and identification of bacteria is dependent on cultivation and isolation and rely on phenotypic- and genotypic-based descriptions [1]. Within the complexity of bacterial diversity, analyses of bacteria typically require combinations of different methods to generate reliable identifications of species. In cases of characterising pathogenic bacteria in clinical samples and diagnosing infectious diseases, these protocols require hours or days, before targeted treatments may be initiated [2]. Such practices lead to risk of increased morbidity and mortality, as well as contributing to rising antibiotic resistance, through preliminary treatment with ineffective antimicrobial agents. The introduction of methods, such as polymerase chain reaction (PCR) and next-generation sequencing (NGS) technologies, have enabled molecular-based identifications and rapid diagnostics in the clinic [3]. In particular, the resolution and high-throughput capabilities of the recent sequencing platforms have facilitated genome-wide characterization and elucidation of clinically-relevant features in a wide range of bacteria [4]. However, genome sequence data, while detecting potential genotypic traits, do not provide information about the features actually being expressed by bacteria, e.g., virulence, antibiotic resistance or responses to environmental dynamics or defence mechanisms of a host organism, which means that complementary protocols, including time-consuming cultivation and characterization steps, are still often necessary.

Another issue related to cultivation-dependent protocols is that many bacteria are not readily recovered and isolated, particularly in cases of clinical samples. The pathogen often may not be isolated in cases of infection; for example, more than 50% of infections caused by *Streptococcus pneumoniae* may not be confirmed by isolation of the bacteria [5]. The importance of being able to differentiate and identify bacteria is essential for understanding bacteria and their activities. The ability to distinguish *S*. *pneumoniae* from other species of the genus *Streptococcus* is highlighted by the recognition that acute respiratory infection (ARI) and pneumonia is the leading cause of death in children under 5 years of age, worldwide [6]. *S*. *pneumoniae* (pneumococcus) is the predominant cause of pneumonia, also with risk of developing into invasive infections and sepsis [7]. However, many streptococci are non-pathogenic, often being found among the commensal human microbiota, and also are often difficult to cultivate and identify, thereby presenting problems for labs to detect and distinguish pathogenic and non-pathogenic strains. Furthermore, dependency on cultivation adds significant bias to bacterial detection and characterisation analyses with risk of skewed and false negative results. Such observations emphasize the importance of cultivation-independent and objective analytical developments for characterizing bacteria, ultimately based upon genome sequence data.

The “proteome” [8] of an organism can be viewed as a “snapshot” of the genome that is being expressed or modified at a given time-point and a given condition, highlighting the functionality of the organism (phenotype). Recent developments in MS instrumentation, including the evolution of Orbitraps [9], have provided the means for rapid, accurate and sensitive detection and characterisation of proteins and peptides.”Proteotyping”, using a “bottom-up” proteomics strategy [10], relying on species-unique or associated peptides for the detection, characterisation and identification of bacteria [11–14], provides the potential for a sensitive, cultivation-independent analytical approach, based upon an integrated strategy dependent upon accurate and reliable genome sequence data, able to obtain deep systematic and functional information on bacterial strains and able to cope with complex mixtures of microorganisms. The capability for detecting thousands of peptides from a single MS analytical run enables simultaneous identification of hundreds or thousands of biomarkers for species- or, even, strain-level determinations, as well as expressed features, providing information about metabolic pathways, virulence, antibiotic resistance, etc. [13].

In this study, we used tandem MS bottom-up proteomics-based analyses for the differentiation and identification of bacteria, i.e., proteotyping, to differentiate the three most closely related species within the Mitis Group of the genus *Streptococcus*: *S*. *pneumoniae*, *S*. *pseudopneumoniae* and *S*. *mitis*. These three species were selected because of their clinical relevance; *S*. *pneumoniae* being a major pathogen, causing deaths on a global scale due to serious infections, including pneumonia, meningitis and sepsis, and also because traditional methodologies used by clinical microbiology laboratories are failing in differentiating these species, in particular. The Type species of the genus, *S*. *pyogenes*, was also included in the study, as a taxonomically distant out-group. The species included in the study were chosen to illustrate the high-resolution differential power of the proteotyping approach, particularly when investigating complex samples that may contain multiple species that are phylogenetically and taxonomically close.

## Materials and methods

### Bacterial strains and cultivation

Reference strains, including the type strains of bacterial species analysed were obtained from the Culture Collection University of Gothenburg (CCUG) (www.ccug.se): *S*. *pneumoniae* strains CCUG 28588^T^, CCUG 7206 and CCUG 35180; *S*. *pseudopneumoniae* strains CCUG 49455^T^, CCUG 62647, CCUG 63747; *S*. *mitis* strains CCUG 31611^T^, CCUG 63687, and CCUG 69183; and *S*. *pyogenes* CCUG 4207^T^, CCUG 25570, CCUG 47803, were cultivated on Blood Agar medium of Columbia Agar Base plus 5% horse blood at 37°C, with 5% CO_2_, overnight. Bacterial biomass was collected and suspended in phosphate-buffered saline 1x (PBS). Bacterial cell suspension optical densities (OD) were measured at a wavelength of 600 nm and adjusted in 1 ml PBS to OD_600_ = 0.8 (10^9^ cfu/ml). The bacterial biomass was washed with PBS three times by centrifuging the sample for 5 min at 12,000 x g, discarding the supernatant and resuspending the pellet in 1.0 ml PBS. Finally, cells were resuspended in 150 μl of PBS and transferred to small vials (200 μl) containing glass beads (G1145, Sigma-Aldrich, St Louis, MO, USA), in preparation for cell lysis, by bead-beating, using a TissueLyser (Qiagen, Hilden, Germany; settings: frequency 25 Hz for 5 min). The bacterial lysates were frozen at -20°C until analysis.

### Peptide generation

To digest bacterial proteins into peptides, the cell lysate was injected into a LPI Hexalane FlowCell (Nanoxis Consulting AB, Gothenburg, Sweden, www.nanoxisconsulting.com; Patent Application No. WO2006068619), using a pipette to fill the FlowCell channel (channel volume of approximately 30 μl). Proteins were immobilized to the FlowCell surface, after incubation for 1 h, at room temperature. The FlowCell channels were washed with 400 μl of ammonium bicarbonate, using a syringe pump, with a flow rate of 100 μl/min. Enzymatic digestion of the proteins was performed by injecting trypsin (2 μg/ml in 20 mM ammonium bicarbonate, pH 8.0) into the FlowCell channels and incubating for 1 h at room temperature. The generated peptides were eluted by injecting 200 μl ammonium bicarbonate buffer (20 mM, pH 8.0) into the channels. The eluted peptides were collected at the outlet ports, using a pipette, and transferred into Axygen tubes (2.0 ml). The peptide solutions were incubated at room temperature overnight and subsequently frozen at −20°C until analysis by MS.

### Liquid chromatography tandem-mass spectrometry (LC-MS/MS) analysis

The tryptic peptides were desalted on PepClean C18 spin columns (Thermo Fisher Scientific, Inc., Waltham, MA, USA), according to the manufacturer’s guidelines, dried and reconstituted with 15 μl of 0.1% formic acid (Sigma Aldrich) in 3% gradient grade acetonitrile (Merck KGaA, Darmstadt, Germany). A sample injection (2 μl) was made with an Easy-nLC autosampler (Thermo Fisher Scientific, Inc.) and analyzed with an interfaced Q Exactive hybrid mass spectrometer (Thermo Fisher Scientific, Inc.). The peptides were trapped on a precolumn (45 x 0.075 mm i.d.) and separated on a reversed phase column, 200 x 0.075 mm, packed in-house with 3 μm Reprosil-Pur C18-AQ particles (Dr. Maisch, Ammerbuch, Germany). The nanoLC (liquid chromatography) gradient was running at 200 nl/min, starting at 7% acetonitrile (ACN) in 0.2% formic acid, increased to 27% ACN for 25 min, then increased to 40% for 5 min and finally to 80% ACN for 5 min and held at 80% ACN for 10 min. Ions were created and sprayed into the mass spectrometer under a voltage of 1.8 kV and capillary temperature of 320°C in data-dependent positive ion mode. Full scan (MS1) spectra were acquired in the Orbitrap over the m/z range, 400–1600, with charge range 2–6 at a resolution of 70,000, until reaching an Automatic Gain Control (AGC) target value of 1*10^6, at a maximum of 110 msec. MS/MS spectra were acquired, using higher energy collision dissociation (HCD) at 30% from m/z 110 for the ten most abundant parent ions, at a resolution of 35,000, using a precursor isolation window of 2 Da, until reaching an AGC target value of 1*10^5 during an injection time of 110 msec. In order to allow for detection of as many peptides as possible, a dynamic exclusion step of the ionized precursor peptides was employed for 30 secs after initial selection for MS/MS.

The liquid chromatography tandem-mass spectrometry (LC-MS/MS) output was converted from the proprietary Thermo/XCalibur RAW format to the open source mzXML format [15], using ReAdW [16] (version 201411.xcalibur), with command line arguments: “—nocompress—gzip". The X! Tandem mass spectra search engine (version VENGEANCE 2015.12.15) [17, 18], was used to identify peptides from the mass spectra data, with the following settings: fragment monoisotopic mass error = 20 mmu; parent monoisotopic mass error plus = 5 mmu; parent monoisotopic mass error minus = 5 mmu; fragment mass type monoisotopic; dynamic range = 100.0; total peaks = 50; maximum parent charge = 4; minimum parent m+h = 800.0 Da; minimum fragment mz = 100.0; minimum peaks = 15; potential modification mass = 16.0@M; maximum valid expectation value = 1.0 [19]. The protein database used to match spectra to peptides was a FASTA file, consisting of 56,967,781 non-redundant proteins from the NCBI GenBank NR [20] database and 6,320,906 peptide sequences from the reference genomes produced by the Human Microbiome Project [21]. All duplicate sequences, as well as sequences containing unidentified peptides (“X”), were removed. The resulting database used with X! Tandem contained a total of 59,349,300 distinct protein sequences.

### TCUP and peptide matching

The in-house developed bioinformatics pipeline, “**T**yping and **C**haracterization **U**sing **P**roteomics” (TCUP), assigned peptides to their lowest unambiguous taxonomic rank, using an implementation of the lowest common ancestor (LCA) algorithm [22], and produced a taxonomic profile, which can be summarized at any given taxonomic level [19]. The application of the TCUP pipeline is divided into two separate steps: in the first step, the generated peptides are matched to a reference database containing complete bacterial genome sequences assembled from NCBI RefSeq [19, 23, 24]; in the second step, peptides that passed the first step are assigned nodes in a taxonomic tree derived from a cluster analysis, based on the NCBI Taxonomy [25]. From this procedure, complete taxonomic profiles and lists of species-unique peptides (i.e., discriminative peptides) are generated by the TCUP pipeline. For the second step in TCUP, the taxonomic assignments of identified peptides, two local databases of reference genome sequence data were created and used in this study. These databases were built and curated from sequences of reference genomes, in FASTA format. During analyses, the genome sequences were translated into the six open reading frames, generating all possible amino acid sequences, against which the detected peptides were matched [19]. The first local database (termed “Initial Database”) contained NCBI RefSeq [26, 27] bacterial genomes (2,785 genomes, downloaded Nov. 17, 2015), with some modifications: sequences that move horizontally between organisms (i.e., mobile genetic elements), potentially appearing in multiple distantly related genomes, were removed; as well as sequences shorter than 400,000 nucleotides; sequences annotated with any of the keywords “plasmid,” “phage,” “extrachromosomal,” “incision element,” or “transposon” were excluded; additionally, genomes annotated as belonging to *Shigella* spp. were also excluded, due to their similarity and taxonomic uncertainty in relation to *Escherichia coli* [28]. The second local reference genomes database (termed “Curated Database”) was based on the “Initial Database” but was augmented by: 1) correcting taxonomic classifications of incorrectly classified reference genomes [26, 27] of *Streptococcus* species included in the Mitis Group, as described by Jensen et al. [29]; 2) adding additional reference genome sequences of species of the *Streptococcus* Mitis Group (S1 and S2 Tables).

During evaluation of the TCUP results, only the matches from a minimum cut-off of five peptide matches to a given species were included in the analyses (i.e., if the genome sequences of a given species were matched by 4 or less peptides from a sample, it was not considered to be a positive identification). The cut-off was determined by evaluating the results from samples containing low numbers of bacteria and, thus, low numbers of species-unique peptides. As demonstrated previously [19], TCUP can estimate the relative abundances of species in a mixture by normalizing the output result with correction factors reflecting expected proportions of unique peptides for the species in a mixture. For this study, the correction factors were determined from analysis of pure cultures of individual representative species, although they also can be predicted by *in-silico* analysis of the proteomes of the species.

### DNA extraction, whole-genome sequencing and assembly

Bacterial genomic DNA was isolated from pure-culture, fresh biomass, using a Wizard Genomic DNA Purification Kit (Promega, Madison, WI, USA), the MagNA Pure Compact Nucleic Acid Isolation Kit I (Roche Diagnostics, Mannheim, Germany) or following a modified version [30] of a previously described protocol [31]. Isolated genomic DNA was sequenced, using the Illumina MiSeq or Illumina HiSeq 2500 platform. Sequencing paired-end reads were assembled, using the CLC Genomic Workbench (versions 8.0.1–10.0.0). The assembled genome sequences were deposited at DDBJ/ENA/GenBank. The details of each genome sequence are presented in the S2 Table. The draft genome sequence of the strain *S*. *mitis* CCUG 69183 (= SK271), was obtained from the NCBI RefSeq database [32].

### Whole-genome sequence average nucleotide identity (ANI) similarity determinations

Genomic affiliations of bacterial strains were assessed by calculations of average nucleotide identity (ANI) similarities. ANI values were calculated, based on the BLAST algorithm (ANIb) [33], by pair-wise genomic comparisons between all genome sequences, using the JSpeciesWS online service [34]. The obtained matrix was used to generate a dendrogram, using PermutMatrix software, applying hierarchical clustering, using average linkage (UPGMA) and Pearson's distance correlation [35]. ANIb calculations were performed for all genome sequences of *S*. *pneumoniae*, *S*. *pseudopneumoniae* and *S*. *mitis* present in the”Curated Database”, and including the genome sequences of the type strains of all species belonging to the Mitis Group of the genus *Streptococcus*, and the type strain of *S*. *pyogenes*, as well as the 12 reference strains of this study. The list of genome sequences included in the Curated Database is available in S2 Table and the information about the twelve proteotyping strains is available in S3 Table.

### Core-genome analysis

The same genome sequences included in the ANI analyses were compared by core genome analysis. All genome sequences were annotated with the open source Prokka software tool version 1.11 [36]. Protein-coding sequences were identified, using Prodigal version 2.3.6 [37]. The amino acid sequences obtained were compared, using the software package, GET_HOMOLOGUES [38], and the derived protein amino acid sequences were compared, using the criterion of 50/50, i.e., 50% similarity for, at least, 50% coverage of alignments. With this criterion, a consensus core genome of single-copy genes was determined using three clustering algorithms (BDBH, COGtriangles and OrthoMCL). The amino acid sequences of the core genome single-copy gene sequences were aligned, using Clustal Omega [39]; poorly aligned regions were removed with Gblocks [40]. The resulting alignments were concatenated, using an in-house pipeline, and phylogenetic clusters were constructed, using the PhyML program [41], applying the approximate likelihood-ratio test for branching statistical support [42]. The phylogenetic tree was visualized, using the Interactive Tree Of Life (iTOL) [43] webserver.

## Results and discussion

### Proteotyping of the representative bacterial strains

**Fig 1:**
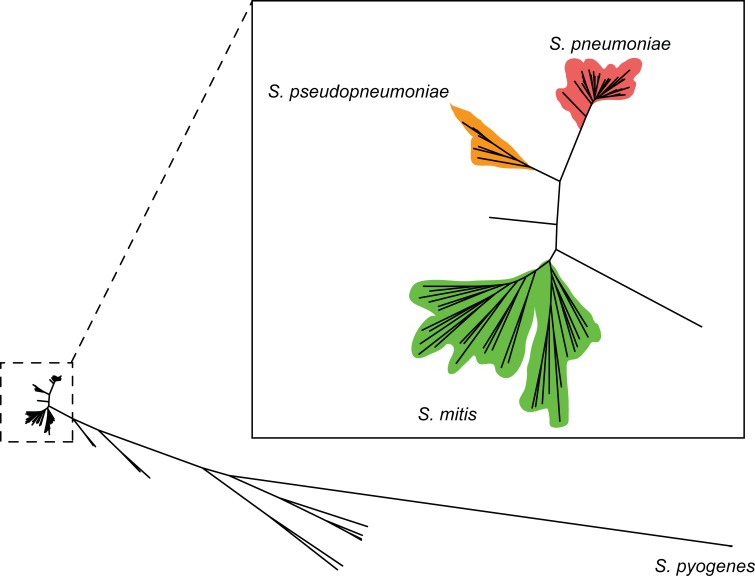
Phylogenetic tree based on core genome analysis of the strains included in the “Curated Database”. The tree includes all strains of *S*. *pneumoniae*, *S*. *pseudopneumoniae*, *S*. *mitis*, including the experimental strains used in the study, as well as the type strain of the other species of the *Streptococcus* Mitis Group included in the database and *S*. *pyogenes*. The tree is based on 168,439 homologous amino acid positions and was constructed, using PhyML software and the aLRT algorithm.

The three most closely-related species of the Mitis Group of the genus *Streptococcus* (i.e., *S*. *pneumoniae*, *S*. *pseudopneumoniae* and *S*. *mitis*), were analyzed, as a model system, to demonstrate the high-resolution differential power of the proteotyping approach. To confirm the phylogenetic affiliations of the twelve representative strains, the genomes of strains were sequenced, assembled and annotated (S3 Table). In the “Curated Database”, the genome sequences belonging to the Mitis Group of the genus *Streptococcus* were selected, according to the recent taxonomic assessment proposed by Jensen et al. [29]. Core genome-based analyses and ANIb calculations, using all pairwise comparisons were performed and the twelve strains were confirmed to belong to their respective species, since the genome sequence ANIb values against their type strains were greater than 95%. The core-genome phylogenetic tree (based on 168,439 amino acid homologous positions) clearly showed that the species *S*. *pneumoniae*, *S*. *pseudopneumoniae* and *S*. *mitis* are extremely closely-related, observing three different clusters (Fig 1). Using the results of the all-strains-versus-all-strains ANIb comparisons, including the twelve proteotyping strains, a dendrogram was constructed (S1 Fig), showing that indeed they belonged to the correct species. Importantly, the whole genome sequences for the twelve representative experimental strains were not added to the genome sequence database used for matching the peptides, in order to test the accuracy of proteotyping for identifications of strains when they are not included in the genome databases, i.e., simulating situations of analyzing isolates obtained directly from samples.

MALDI-TOF MS (VITEK MS, bioMérieux SA, Marcy l´Etoile, France) identifications were performed on the strains included in this study, by submitting them to a hospital Clinical Microbiology Laboratory for routine processing, according to accredited protocols (data not shown). MALDI-TOF MS profiling was able to differentiate the species *S*. *pyogenes*, *S*. *pneumoniae* and *S*. *pseudopneumoniae*. Even though it was possible to differentiate these species, using MALDI-TOF MS, *S*. *mitis* was not able to be identified at the species-level, but was classified as “*S*. *mitis*/*S*. *oralis* complex”. Although the current commercial VITEK MS V3.2.0 IVD CE-marked database for MALDI-TOF MS was observed to have been improved from past versions, bacterial identifications are still recognised to be dependent on initial isolation steps and obtaining pure cultures of the strains to be identified; the methodology is not effective for analysing samples from which the bacteria are not isolated or for those containing multiple species. Proteotyping is able, not only to correctly identify all strains of the species in this study, including differentiating *S*. *mitis* from *S*. *oralis* by more than 250 species-unique peptides (S13 Table), but may also be applied for cultivation-independent analyses, applicable to samples containing complex mixtures of different species, making it suitable for direct analyses of samples, including those from clinical infections.

The twelve representative experimental strains were analysed in triplicate using the Lipid-based Protein Immobilization (LPI) methodology, which has been shown in several studies to promote the detection of species-unique peptides [12, 13, 44]. The number of proteins and peptides, including the species-unique peptides for each of the representative strains are shown in Table 1.

**Table 1:** Proteotyping results of the twelve representative strains included in the study. For each species, the Type strain, as well as two additional well-characterized reference strains, were included. The numbers of identified proteins, peptides and species-unique peptides after analyses with TCUP are shown (averages of triplicate analyses). The accuracies (%), i.e. proportion of correctly assigned peptides of the total number of species-unique peptides, are also shown. For a confirmed identification, a minimum threshold of five peptide matches per species was used.

Organism	Strain	Distinct proteins	Peptide matches	Species-unique peptide matches	Accuracy (%)
*S*. *pneumoniae*	CCUG 28588^T^	590	3188	227	**97**
	CCUG 7206	590	2251	175	**100**
	CCUG 35180	600	2384	214	**100**
*S*. *mitis*	CCUG 31611^T^	610	3418	272	**100**
	CCUG 63687	660	2542	287	**100**
	CCUG 69183	479	3478	506*	**99**
*S*. *pseudopneumoniae*	CCUG 49455^T^	611	2743	433	**100**
	CCUG 62647	574	2450	257	**97**
	CCUG 63747	524	2082	245	**100**
*S*. *pyogenes*	CCUG 4207^T^	401	1290	314	**100**
	CCUG 25570	450	1801	351	**100**
	CCUG 47803	493	2002	418	**100**

* The genome sequence of CCUG 69183 (= SK271) was present in the “Curated Database”, which is reflected by the higher number of species-unique peptide matches.

^T^ signifies type strain of the species.

The data in Table 1 shows the accuracies of matching peptides with the correct species for the strains of the species *S*. *pneumoniae*, *S*. *pseudopneumoniae* and *S*. *mitis*, as well as the three strains of *S*. *pyogenes*, when matching the peptides against the “Curated Database” (i.e., after addition of reference genome sequences and manual curation). The accuracy (%), calculated from the triplicate analyses of each strain, is the percentage of correctly assigned peptide matches, in relation to the total number of species-unique peptide matches. When using a cut-off of a minimum of five peptide matches per species as a positive identification, the noise level, i.e., random, non-specific peptide matches, were reduced and the accuracy for species identifications was increased [19]. The accuracies of peptide matching were high, using the “Curated Database”, which had been supplemented by adding the genome sequences of reference strains and by controlling the quality of the identifications of the genome sequence data.

From the proteotyping analyses of the twelve experimental strains, and after confirming that the identified peptides were matched to their respective species, the species-unique peptides could be linked to the proteins from which they originated. For example, the three MS analyses of the Type strain of *S*. *pneumoniae*, CCUG 28588^T^, identified as many as 25 species-unique peptides linked to the same protein, resulting in amino acid sequence coverage of almost 50% (Table 2 and S4–S12 Tables).

**Table 2:** List of proteins identified from the species-unique peptides. Peptides detected and identified in triplicate analysis of one of the twelve experimental strains, *S*. *pneumoniae* CCUG 28588^T^,were linked to their respective proteins. Only proteins having two or more peptide matches are shown here. Full lists of proteins and peptides for the representative strains of *S*. *pneumoniae*, *S*. *pseudopneumoniae* and *S*. *mitis* included in the study can be found in S4–S12 Tables.

Accession number	Description	Number of peptides	Sequence coverage
WP_001035310.1	hypothetical protein	25	47%
WP_000685088.1	UDP-glucose dehydrogenase	11	44%
WP_001844726.1	endo-beta-N-acetylglucosaminidase	10	9%
WP_065251743.1	choline-binding protein	10	23%
WP_000679960.1	beta-N-acetylhexosaminidase	8	8%
WP_001214397.1	NAD-dependent dehydratase	7	24%
WP_000727933.1	Foldase	6	24%
WP_000434652.1	thiol reductase thioredoxin	4	44%
WP_000495824.1	transcriptional regulator	4	43%
WP_000811753.1	alanine—tRNA ligase	4	4%
WP_000036661.1	dihydroxyacetone kinase	3	9%
WP_000064115.1	general stress protein	3	24%
WP_000767195.1	hypothetical protein	3	13%
WP_000862350.1	glycosyl transferase family 1	3	10%
WP_001079795.1	galacturonic acid acetylase	3	22%
WP_001818788.1	PTS glucose transporter subunit IIABC	3	8%
WP_000116461.1	trigger factor	2	8%
WP_000164758.1	glycine—tRNA ligase subunit beta	2	4%
WP_000201902.1	RNA polymerase sigma factor SigA	2	5%
WP_000245505.1	30S ribosomal protein S8	2	14%
WP_000411198.1	choline kinase	2	10%
WP_000432756.1	alpha-mannosidase	2	5%
WP_000529016.1	serine protease	2	4%
WP_000599104.1	ribosome-associated factor Y	2	6%
WP_000639574.1	hypothetical protein	2	43%
WP_000664173.1	capsular polysaccharide biosynthesis CpsC	2	16%
WP_000701442.1	PTS fructose transporter subunit IIC	2	5%
WP_000790743.1	hypothetical protein	2	16%
WP_001032504.1	YSIRK signal domain/LPXTG anchor	2	2%
WP_001092741.1	arginine—tRNA ligase	2	6%
WP_001162938.1	dihydrolipoyl dehydrogenase	2	5%
WP_001229596.1	arginine ABC transporter ATP-binding	2	15%
WP_001232820.1	alkaline amylopullulanase	2	4%
WP_001818543.1	cell division DivIVA	2	11%

**Fig 2:**
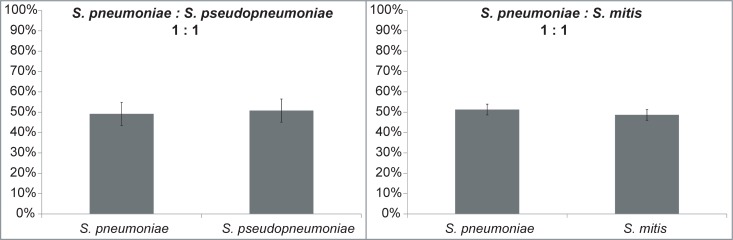
Proteotyping results of mixed samples. Cells of *S*. *pneumoniae* and *S*. *pseudopneumoniae* or *S*. *mitis* were mixed in ratios of 1:1. Following sample preparation, digestion and LC-MS/MS analyses, the results were evaluated, using pre-computed correction factors, reflecting the expected proportion of unique peptides of each of the species. In both mixes, *S*. *pneumoniae*:*S*. *pseudopneumoniae* and *S*. *pneumoniae*:*S*. *mitis*, the results reflected a composition of approximately 50% of each species (standard error bars on averages from triplicate analyses).

As it is possible to find the three species of the *Streptococcus* Mitis Group included in this study in the same clinical sample, bacterial cells from *S*. *pneumoniae* CCUG 28588^T^ were mixed with cells from *S*. *pseudopneumoniae* CCUG 49455^T^ or *S*. *mitis* CCUG 31611^T^ in 1:1 mixtures, to assess the capability of proteotyping to detect and identify species-unique peptides in simple experimental mixed sample (Fig 2). The experiments showed that proteotyping could accurately distinguish between the species in the mixed samples. For the mixture of *S*. *pneumoniae* and *S*. *pseudopneumoniae*, the relative abundances were estimated to 49% and 51%. For *S*. *pneumoniae* and *S*. *mitis* the corresponding numbers were 51% and 49%.

### Considerations for performing proteotyping

**Fig 3:**
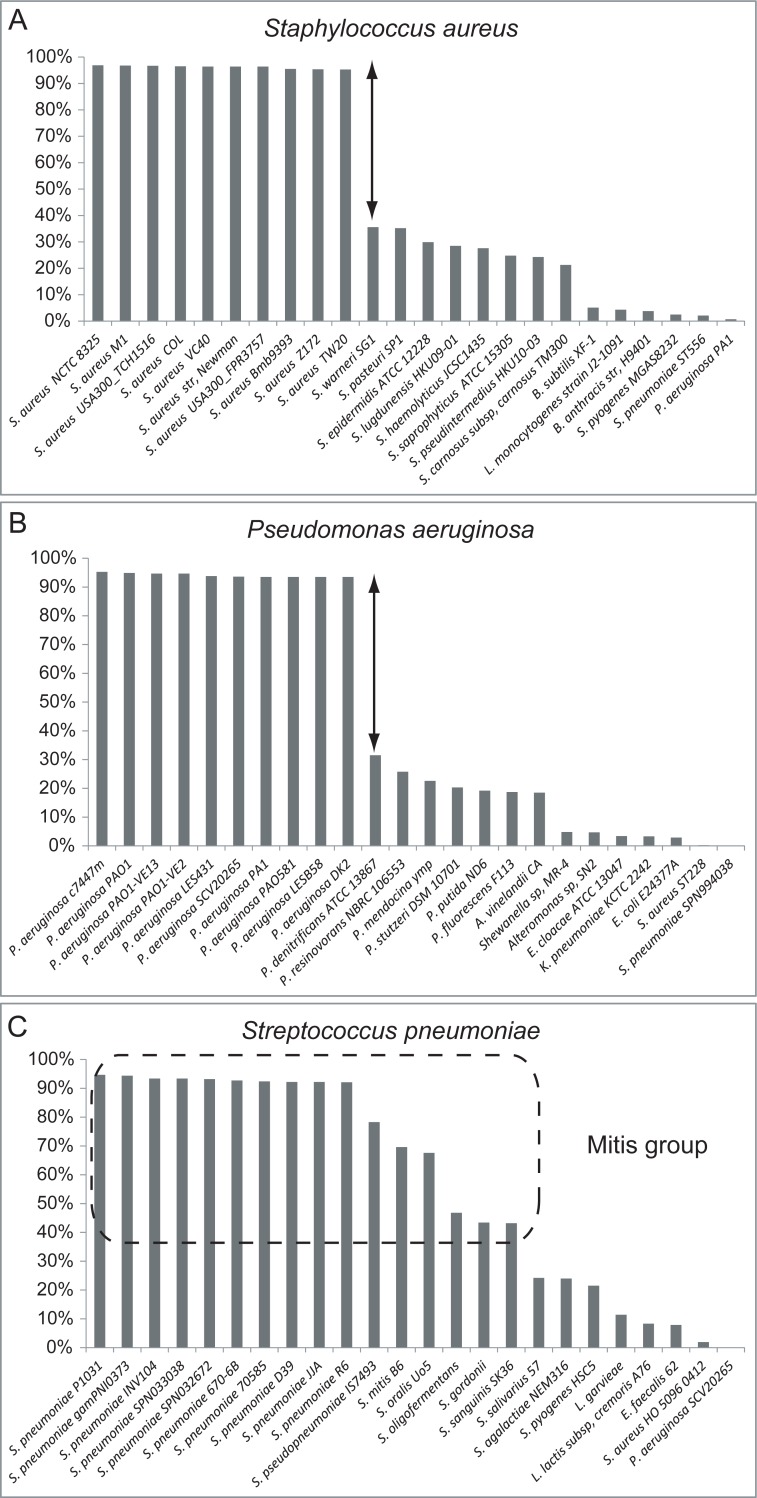
Ranking according to matching efficiency (%) of the identified peptides against complete genome sequences from RefSeq database. Proteotyping results, following MS-proteomic analyses of strains of *S*. *aureus* (A), *P*. *aeruginosa* (B) and *S*. *pneumoniae* (C). For *S*. *aureus* and *P*. *aeruginosa*, the top ranked peptide matches are all with the correct species in the RefSeq database. The next-best ranked matches are for other species of *Pseudomonas* and *Staphylococcus*, marked with arrows, reflecting a distinct drop from almost 100% down to 30% in matching efficiencies of identified peptides. From the analysis of *S*. *pneumoniae* (C), the top ranked matches all belong to the correct species (*S*. *pneumoniae*), although, due to the phylogenetic relationships and taxonomy of this species in the *Streptococcus* genus, the matching efficiencies to other species, especially for *S*. *pseudopneumoniae* and *S*. *mitis*, is relatively higher (as much as 70–80%), thus making discovery of species-unique peptides more difficult.

The use of peptides for identifications and characterisations of bacteria, i.e., bottom-up proteomics-based proteotyping, has been applied in limited numbers of earlier studies [12, 45, 46]; for peptide biomarkers discovery for *Bacillus anthracis* [47, 48], *Mycobacterium tuberculosis* [49], *Staphylococcus aureus* [50], *Acinetobacter baumannii* [51, 52] and the respiratory tract pathogens *Acinetobacter baumannii*, *Moraxella catarrhalis*, *Pseudomonas aeruginosa*, *Stenotrophomonas maltophilia*, and *Klebsiella pneumoniae* [53]. An important aspect to keep in mind when applying proteotyping is that the discriminatory power is strongly linked to the taxonomic relationships among different bacterial species. This is due, in large part, to the dependency on the relationships between genome sequences via the taxonomic hierarchy and the ability to determine species-unique peptides, using the LCA algorithm of TCUP [19]. Species that are easily differentiated and identified, with relatively large phylogenetic distances and taxonomic differences to other species, such as *Pseudomonas aeruginosa* or *Staphylococcus aureus*, provide high numbers of species-unique peptides, separating them from their closest relatives. When analysing peptides from strains of *Staphylococcus aureus* and *Pseudomonas aeruginosa* and then matching the identified peptides to the available genome sequences in the NCBI Reference sequence database [26, 27], the matching efficiencies against the available genome sequences for these species was observed to be high (> 90%). The taxonomically closest, albeit relatively distantly related species did not show comparatively high levels of sequence matching (approximately 30%) (Fig 3A and 3B).

In contrast, bacterial species that are phylogenetically more closely related become more difficult to differentiate from each other since the number of peptides that are shared will be higher. Consequently, the number of species-unique peptides is reduced, which is the case for the species of the genus *Streptococcus*, particularly including species of the Mitis Group. This was clearly shown in our analysis. For the type strain of *S*. *pneumoniae*, all top-ranked peptide-matching genomes with the highest number of matching peptides belonged to the correct species (>90% matching efficiency) (Fig 3C). However, the similarity gap of peptide-matching to the genomes from closely related species, including *S*. *pseudopneumoniae* and *S*. *mitis* (approximately 80% matching efficiency), was substantially less. This illustrates the difficulty associated with finding species-unique peptides and differentiating between taxonomically-similar species.

### Considerations of the reference genome database

Proteotyping is highly dependent on access to accurate and comprehensive genome sequence databases, including as many species as possible, as well as sufficient numbers of strains to represent the genomic diversity of each species. In most cases, a single genome does not satisfactorily cover the full genetic variability present within a bacterial species. This is reflected in the species “pan-genome” concept (i.e., the entire pool of genes that are present in different strains of the species), which considers a given species “core genome” (i.e., genes that are shared by the majority of the strains of the species) as well as the species “accessory genome” (i.e., genes that are present in one or several strains of a species, but not in all) [54, 55]. In the case of *S*. *pneumoniae*, several studies have shown that the majority of genes of the pan-genome are absent in many strains, suggesting that the species heterogeneity is relatively substantial [56, 57]. For such reasons, it is important to include genome sequences of multiple strains of each species in the reference database; otherwise, peptides may be incorrectly assigned. Furthermore, peptides expressed by genes not present in the database will not be matched and are, thus, unavailable for proteotyping analyses. More critically, peptides that, in reality, are shared between species, but are assigned to a single species, because the other species may not be represented in the database, can be erroneously reported as species-unique and may, thus, contribute to incorrect interpretations. Fortunately, recent developments in NGS technologies have enabled cost-efficient generation of genome sequence data for numerous bacteria, although still more effort needs to be focused on sequencing more species diversity, including type strains and other important reference strains.

Another related issue is the high number of misclassifications among the genomes archived in public databases [58, 59]. This is particularly true for the genome sequences of strains identified as species of the *Streptococcus* Mitis Group, wherein a recent study observed numerous genome sequences with incorrect taxonomic affiliations [29]. When applying proteotyping analyses, it is critical that the genome sequence database used does not include misclassified genomes, or else the number of species-unique peptides could be markedly reduced. If the genome sequence of a species is misclassified, peptides that are unique for the species, may appear as shared between species, markedly decreasing the number of species-unique peptides that can be exploited as reliable biomarkers. It is therefore critically important to use correctly curated genome sequence databases and to confirm the taxonomic affiliations of the sequence data. Numerous methods have been developed that may be applied for database genome sequence data quality control, such as the average nucleotide identity (ANI) [33, 60], which is implemented in several publicly available stand-alone and on-line software, for example, the software JSpecies [61] and the on-line server JSpeciesWS [34].

The genomes of important human, animal and plant pathogens are usually well-represented in reference sequence databases, such as the DDBJ/ENA/GenBank and PATRIC [62]. However, this contrasts with the many commensal species and environmental species that are less well-studied and typically are underrepresented, even though they may be evolutionarily closely related to pathogenic species. When using sequence data in comparative analyses, imbalances of representative genome sequences, reflecting the genomic variabilities of species, may lead to false-positive species-unique peptide identifications. To illustrate the importance of the database when matching the proteotyping data, analyses were performed, using a reference database containing only the complete genomes of *S*. *pneumoniae*, *S*. *pseudopneumoniae* and *S*. *mitis* that were available from the NCBI (downloaded Nov 2015). In total, the database included 35 complete genomes from the *Streptococcus* Mitis Group (Table 3, “Initial Database”), wherein *S*. *pneumoniae*, *S*. *pseudopneumoniae* and *S*. *mitis* were represented by 27, 1 and 1 genomes, respectively. For many of the other species of the Mitis Group, no genome sequences were available (Table 3).

**Table 3:** Lists of genomes of species of the Mitis Group of the genus *Streptococcus*, used for matching the proteomic data at two different time points. Two databases were used, created in February 2015 (“Initial Database”) and August 2016 (“Curated Database”). The (T) denotes the presence of the Type strain genome of a given species in the database.

Organism	“Initial Database” Before addition of reference genomes	“Curated Database” After addition of reference genomes
*Streptococcus australis*	0	1 (T)
*Streptococcus cristatus*	0	1 (T)
*Streptococcus gordonii*	1	3 (T)
*Streptococcus infantis*	0	1 (T)
*Streptococcus mitis*	1	30 (T)
Streptococcus oligofermentans*	1 (T)	1 (T)
*Streptococcus oralis+*	1 (T)	13 (T)
*Streptococcus parasanguinis*	2 (T)	3 (T)
*Streptococcus peroris*	0	1 (T)
*Streptococcus pneumoniae*	27	31 (T)
*Streptococcus pseudopneumoniae*	1	6 (T)
*Streptococcus sanguinis*	1	6
*Streptococcus sinensis*	0	1 (T)
Streptococcus tigurinus*	0	4 (T)
Sum of Mitis Group genomes	35	102
Ψ *Streptococcus pyogenes*	19	47

*S. *oligofermentans* is considered to be a later heterotypic synonym of *S*. *cristatus* and *S*. *tigurinus* is considered to be a subspecies of *S*. *oralis* [29].

+*S oralis* subsp. *tigurinus* genomes, previously classified as *S*. *tigurinus*, are included as *S*. *oralis*, according to Jensen et al., 2016 [29]. The reference strain for the subspecies, *S*. *oralis* subsp. *tigurinus* AZ_3a is included in the database.

Ψ *S*. *pyogenes* is included here, as an out-group species, since this species was part of the model system.

To adjust for the imbalance and lack of genetic variation within individual species, additional draft genome sequences from the RefSeq database were analyzed, confirmed to belong to the *Streptococcus* taxonomy proposed by Jensen et al. [29], and subsequently included into the Curated Database. Additionally, in-house genome sequencing was performed, to increase the number of Mitis Group genome sequences included in the database, particularly genome sequences for additional strains of the *S*. *pseudopneumoniae* and *S*. *mitis* species. Furthermore, the genome sequences of the type strains of each species in the Mitis Group according to Jensen et al. [29], were sequenced, analysed, confirmed and included into the Curated Database. Following these genome additions, the “Curated Database” was updated to include 102 genomes belonging to the Mitis Group (Table 3, “Curated Database”). All the genome sequences listed in the “Curated Database”were curated manually and the taxonomic and phylogenetic affiliations assessed, using ANIb pairwise comparisons between all genomes, as well as core genome-based analysis. Genome sequences with ANIb % similarities higher than 95% were considered to belong to the same species. In Table 3, the genome sequence data available in each database (“Initial” and “Curated”) for the analyses of the species of the Mitis Group of the genus *Streptococcus* is indicated.

The results of proteotyping of the twelve representative strains were evaluated by performing two separate analyses, using the “Initial Database” and the “Curated Database” (Table 4). As Table 4 shows, accuracy of peptide identifications can be seen to be unaffected when matching the peptides of *S*. *pneumoniae* and *S*. *pyogenes* against both databases. This is most likely because the number of genome sequences for *S*. *pneumoniae* and *S*. *pyogenes* was already sufficiently high and the genomic variability was well-represented within the species. Therefore, most of the peptides could be correctly matched to available genomes, leading to high accuracy and discriminatory power. When instead, the “Curated Database” was used, all *S*. *pneumoniae* strains exhibited a lower number of species-unique peptides. This is a consequence of the addition of genomes of the closely-related species, *S*. *pseudopneumoniae* and *S*. *mitis* and more accurately describing their within-species genomic heterogeneities. Peptides that were classified as unique for *S*. *pneumoniae* using the “Initial Database” were, with the “Curated Database”, recognized, in fact, as shared between species. This demonstrates that an incomplete database can result in a large number of false positives.

**Table 4:** Proteotyping results of the twelve representative strains included in the study (averages of triplicate analyses). The number of species-unique peptide matches and accuracies (%), using the two databases are shown in the columns headed “Initial Database” and “Curated Database”. A minimum threshold of five peptide matches per species was used in the analysis. The improvement in accuracies for *S*. *mitis* and *S*. *pseudopneumoniae* is highlighted in bold.

Organism	Strain	Initial Database		Curated Database	
		Species-unique peptide matches	Accuracy (%)	Species-unique peptide matches	Accuracy (%)
*S*. *pneumoniae*	CCUG 28588^T^	354	98	227	97
	CCUG 7206	286	100	175	100
	CCUG 35180	380	100	214	100
*S*. *mitis*	CCUG 31611^T^	377	**72**	272	**100**
	CCUG 63687	201	**72**	287	**100**
	CCUG 69183	227	**59**	506	**99**
*S*. *pseudopneumoniae*	CCUG 49455^T^	329	**70**	433	**100**
	CCUG 62647	230	**82**	257	**97**
	CCUG 63747	232	**79**	245	**100**
*S*. *pyogenes*	CCUG 4207^T^	319	100	314	100
	CCUG 25570	360	100	351	100
	CCUG 47803	427	100	418	100

^T^ signifies type strain of the species.

In the case of *S*. *pyogenes*, 19 genomes already were present in the Initial Database, such that the genomic heterogeneity of the species was well-covered. The accuracy of peptide matching for this species was, therefore, high regardless of the databases used. Furthermore, *S*. *pyogenes* is taxonomically more distant to related species, compared to the situation of *S*. *pneumoniae*, *S*. *pseudopneumoniae* and *S*. *mitis*, which reduces the probability for incorrect species identifications and misidentification of species-unique peptides. For *S*. *pseudopneumoniae* and *S*. *mitis*, the added genome sequences in the Curated Database helped adjust the marked imbalances presented in the Initial Database and greatly improved the accuracy of the proteotyping analyses. Proteotyping accuracy, using the Initial Database, was approximately 70%, whereas it was improved to 97–100% when using the Curated Database, better representing the genomic variability of these species. In the cases of the strains of *S*. *pseudopneumoniae*, the analyses, using the Initial Database, showed that most of the incorrectly-matched peptides were assigned to *S*. *pneumoniae*, whereas for *S*. *mitis*, the incorrectly-matched peptides were assigned to *S*. *pneumoniae* and *S*. *pseudopneumoniae* (S2 Fig and S13 Table).

## Discussion

In this study, bottom-up, tandem MS proteomics-based analyses, i.e., proteotyping, was demonstrated to be a reliable, high-resolution methodology for characterizing and identifying bacteria, at the species-level. Using a model system of the most closely related bacterial species of the *Streptococcus* Mitis Group, which typically cause problems for laboratories to detect and identify, proteotyping enabled discovery of more than 250 species-unique peptides for each of the three most closely related species, the pathogen, *S*. *pneumoniae*, and the commensals, *S*. *pseudopneumoniae* and *S*. *mitis*.

An advantage of DNA-based methods for analysing microorganisms is the possibility to amplify a particular marker, using polymerase-chain reaction (PCR)-based methods, for detection of genotypic traits. Furthermore, methods involving PCR or quantitative PCR (qPCR) have been developed, enabling direct and cultivation-independent analyses of samples, easily applied for routine work-flows. For instance, after analyzing more than 600 genome sequences of species of the Mitis Group, the “Xisco” gene was recently described as a reliable biomarker for PCR-based detection and identification of *S*. *pneumoniae* [63]; this tool is currently being applied in clinical laboratory protocols. The main benefit of such approaches is the high level of sensitivity, stemming from the capability of the method to amplify merely a few copies of a target gene or DNA sequence. Drawbacks include the limitation that these methods are necessarily targeted and, thus, are constrained to the detection of only pre-defined genomic regions. An advantage of the proteotyping approach is the “shotgun” detection of expressed genomic traits, i.e., without being restricted to pre-defined bio-marker targets. Furthermore, a non-targeted proteomics approach enhances discovery of novel expressed features. Although, while not as sensitive as PCR-based protocols, proteotyping sensitivity can be increased by employing an MS-mode targeting defined peptides, for example, in parallel reactions monitoring (PRM) [64]. Peptides commonly expressed by many or, preferably, all strains of a given species would be optimal for use as taxonomic biomarkers and peptides associated with antibiotic resistance, virulence or particular metabolic features would be applicable for the detection of important genotypic traits being expressed phenotypically.

Being able to bypass cultivation steps and subsequent time-consuming characterizations of bacterial isolates, enabling direct analyses of samples, proteotyping adds valuable insights into the expressed phenotypes of bacteria (i.e., function), which only can be suggested by genotypic and genomic approaches (i.e., potential). For patients suffering from severe infections, rapid and accurate diagnoses are vital. The proteotyping workflow provides the necessary accuracy and specificity needed for the differentiation and identification of infectious bacteria for accurate diagnostics. With the ability to detect and characterize bacterial pathogens in clinical samples without prior cultivation, proteotyping has potential for significant decrease in the time needed for providing diagnoses. Admittedly, current implementation of tandem mass spectrometry for clinical microbiological laboratories is costly and is not designed for high throughput procedures. However, only within the last decade has MALDI-TOF MS for microbial profiling and identification been implemented in clinical laboratories and now is a central part of the routine clinical laboratory protocols for infectious disease diagnostics. The tremendous gains in speed and sensitivity in tandem MS in the last decade will continue and is expected to pave the way to implementation of proteotyping, together with the utilization of targeted MS analyses, using peptide biomarkers for defined bacterial species. We have demonstrated that bottom-up MS-proteomics-based proteotyping provides an innovative means to more comprehensive bacterial characterizations with many potential applications.

## Supporting information

S1 Table Mitis Group genomes included in the “Initial Database”. (PDF)

S2 Table Mitis Group genomes included in the “Curated Database”. (PDF)

S3 Table Information about the twelve strains used as proteotyping model system. (PDF)

S4 Table List of proteins identified by species-unique peptides in triplicate analysis of *S. pneumoniae* CCUG 28588^T^. (PDF)

S5 Table List of proteins identified by species-unique peptides in triplicate analysis of *S. pneumoniae* CCUG 7206. (PDF)

S6 Table List of proteins identified by species-unique peptides in triplicate analysis of *S. pneumoniae* CCUG 35180. (PDF)

S7 Table List of proteins identified by species-unique peptides in triplicate analysis of *S. mitis* CCUG 31611^T^. (PDF)

S8 Table List of proteins identified by species-unique peptides in triplicate analysis of *S*. *mitis* CCUG 63687. (PDF)

S9 Table List of proteins identified by species-unique peptides in triplicate analysis of *S*. *mitis* CCUG 69183 (SK271). (PDF)

S10 Table List of proteins identified by species-unique peptides in triplicate analysis of *S*. *pseudopneumoniae* CCUG 49455^T^. (PDF)

S11 Table List of proteins identified by species-unique peptides in triplicate analysis of *S*. *pseudopneumoniae* CCUG 62647. (PDF)

S12 Table List of proteins identified by species-unique peptides in triplicate analysis of *S*. *pseudopneumoniae* CCUG 63747. (PDF)

S13 Table Number of peptide matches for S2 Fig., showing the species assignments using the “Initial” vs the “Curated” Databases. (PDF)

S1 Fig Dendrogram showing correct taxonomic affiliation of the twelve proteotyping strain amongst the genomes included in the “Curated Database”. Dendrogram based on an all-vs.-all ANIb analysis of all the genomes of *S*. *pneumoniae* (n = 31), *S*. *pseudopneumoniae* (n = 6) and *S*. *mitis* (n = 30) included in the “Curated database”, plus the type strains of all the other species of the Mitis Group included in the “Curated database”, *S*. *pyogenes*, as well as the twelve representative strains analysed with proteotyping, which are indicated in color (three each from *S*. *pneumoniae* (red boxes), *S*. *pseudopneumoniae* (orange boxes), *S*. *mitis* (green boxes) and *S*. *pyogenes* (blue boxes).
(EPS)

S2 Fig Number of species assignments from peptide matching of the TCUP pipeline for the 12 reference strains, using the “Initial Database” and “Curated Database” databases. Each bar graph represents average values from triplicate analyses. Only hits above a cut-off of five or more hits were included in the analysis. The percentage of correct species assignment is given on each bar. The color code for the species is given below the bars.
(EPS)
