# Supplementary material for: Proteotyping bacteria: Characterization, differentiation and identification of pneumococcus and other species within the Mitis Group of the genus Streptococcus by tandem mass spectrometry proteomics
Source: PLoS One. 2018 Dec 10;13(12):e0208804. doi: 10.1371/journal.pone.0208804 (PMC6287849; doi:10.1371/journal.pone.0208804)
Supplement: S1 Table — (PDF) [file pone.0208804.s001.pdf]

S1 Table. Mitis group genomes included in the Initial Database

Organism	Strain	GenBank Accession number
Streptococcus gordonii	CH1	NC_009785.1
Streptococcus mitis	B6	NC_013853.1
Streptococcus oligofermentans	AS 1.3089	NC_021175.1
Streptococcus oralis	Uo5	NC_015291.1
Streptococcus parasanguinis	ATCC 15912 T	NC_015678.1
Streptococcus parasanguinis	FW213	NC_017905.1
Streptococcus pneumoniae	70585	NC_012468.1
Streptococcus pneumoniae	670-6B	NC_014498.1
Streptococcus pneumoniae	A026	NC_022655.1
Streptococcus pneumoniae	AP200	NC_014494.1
Streptococcus pneumoniae	ATCC 700669	NC_011900.1
Streptococcus pneumoniae	CGSP14	NC_010582.1
Streptococcus pneumoniae	D39	NC_008533.1
Streptococcus pneumoniae	G54	NC_011072.1
Streptococcus pneumoniae	gamPNI0373	NC_018630.1
Streptococcus pneumoniae	Hungary19A	NC_010380.1
Streptococcus pneumoniae	INV104	NC_017591.1
Streptococcus pneumoniae	INV200	NC_017593.1
Streptococcus pneumoniae	JJA	NC_012466.1
Streptococcus pneumoniae	OXC141	NC_017592.1
Streptococcus pneumoniae	P1031	NC_012467.1
Streptococcus pneumoniae	R6	NC_003098.1
Streptococcus pneumoniae	SPN032672	NC_021003.1
Streptococcus pneumoniae	SPN033038	NC_021004.1
Streptococcus pneumoniae	SPN034156	NC_021006.1
Streptococcus pneumoniae	SPN034183	NC_021028.1
Streptococcus pneumoniae	SPN994038	NC_021026.1
Streptococcus pneumoniae	SPN994039	NC_021005.1
Streptococcus pneumoniae	SPNA45	NC_018594.1
Streptococcus pneumoniae	ST556	NC_017769.2
Streptococcus pneumoniae	Taiwan19F	NC_012469.1
Streptococcus pneumoniae	TCH8431	NC_014251.1
Streptococcus pneumoniae	TIGR4	NC_003028.3
Streptococcus pseudopneumoniae	IS7493	NC_015875.1
Streptococcus sanguinis	SK36	NC_009009.1
