# Supplementary material for: Proteotyping bacteria: Characterization, differentiation and identification of pneumococcus and other species within the Mitis Group of the genus Streptococcus by tandem mass spectrometry proteomics
Source: PLoS One. 2018 Dec 10;13(12):e0208804. doi: 10.1371/journal.pone.0208804 (PMC6287849; doi:10.1371/journal.pone.0208804)
Supplement: S2 Table — (PDF) [file pone.0208804.s002.pdf]

S2 Table. Mitis group genomes included in the Curated Database

No.	Organism	Strain	GenBank accession number
1	*Streptococcus pseudopneumoniae*	IS7493	NC_015875.1
2	*Streptococcus pseudopneumoniae*	ATCC BAA-960T	NZ_AICS00000000.1
3	*Streptococcus pseudopneumoniae*	1321	NZ_AYRP00000000.1
4	*Streptococcus pseudopneumoniae*	276-03	NZ_LJHJ00000000.1
5	*Streptococcus pseudopneumoniae*	338-14	NZ_LJHI00000000.1
6	*Streptococcus pseudopneumoniae*	61-14	NZ_LJHK00000000.1
7	*Streptococcus pneumoniae*	R6	NC_003098.1
8	*Streptococcus pneumoniae*	D39	NC_008533.1
9	*Streptococcus pneumoniae*	70585	NC_012468.1
10	*Streptococcus pneumoniae*	JJA	NC_012466.1
11	*Streptococcus pneumoniae*	P1031	NC_012467.1
12	*Streptococcus pneumoniae*	Taiwan19F-14	NC_012469.1
13	*Streptococcus pneumoniae*	Hungary19A-6	NC_010380.1
14	*Streptococcus pneumoniae*	G54	NC_011072.1
15	*Streptococcus pneumoniae*	CGSP14	NC_010582.1
16	*Streptococcus pneumoniae*	ATCC 700669	NC_011900.1
17	*Streptococcus pneumoniae*	AP200	NC_014494.1
18	*Streptococcus pneumoniae*	670-6B	NC_014498.1
19	*Streptococcus pneumoniae*	SPNA45	NC_018594.1
20	*Streptococcus pneumoniae*	TCH8431/19A	NC_014251.1
21	*Streptococcus pneumoniae*	INV200	NC_017593.1
22	*Streptococcus pneumoniae*	OXC141	NC_017592.1
23	*Streptococcus pneumoniae*	INV104	NC_017591.1
24	*Streptococcus pneumoniae*	SPN034156	NC_021006.1
25	*Streptococcus pneumoniae*	SPN034183	NC_021028.1
26	*Streptococcus pneumoniae*	SPN994038	NC_021026.1
27	*Streptococcus pneumoniae*	SPN994039	NC_021005.1
28	*Streptococcus pneumoniae*	SPN032672	NC_021003.1
29	*Streptococcus pneumoniae*	SPN033038	NC_021004.1
30	*Streptococcus pneumoniae*	ST556	NC_017769.2
31	*Streptococcus pneumoniae*	TIGR4 (Tettelin, 2001)	NC_003028.3
32	*Streptococcus pneumoniae*	TIGR4 (Ribeiro, 2012)	NZ_AKVY00000000.1
33	*Streptococcus pneumoniae*	gamPNI0373	NC_018630.1
34	*Streptococcus pneumoniae*	PCS8235	NZ_CM001835.1
35	*Streptococcus pneumoniae*	NT_110_58	NZ_CP007593.1
36	*Streptococcus pneumoniae*	A66	NZ_LN847353.1
37	*Streptococcus pneumoniae*	NCTC7465T	NZ_LN831051.1
38	*Streptococcus mitis*	B6	NC_013853.1
39	*Streptococcus mitis*	KCOM 1350 (= ChDC B183)	NZ_CP012646.1
40	*Streptococcus mitis*	SVGS_061	NZ_CP014326.1
41	*Streptococcus mitis*	NCTC 12261T	NZ_AEDX00000000.1
42	*Streptococcus mitis*	1111_SMIT	NZ_JWCV00000000.1
43	*Streptococcus mitis*	11-5	NZ_AQTT00000000.1
44	*Streptococcus mitis*	13/39	NZ_AQTU00000000.1
45	*Streptococcus mitis*	17/34	NZ_ASZZ00000000.1
46	*Streptococcus mitis*	18/56	NZ_ATAA00000000.1
47	*Streptococcus mitis*	21/39	NZ_AYRR00000000.1
48	*Streptococcus mitis*	850_SMIT	NZ_JUQO00000000.1

49	*Streptococcus mitis*	OT25	NZ_JYGP00000000.1
50	*Streptococcus mitis*	SK1073	NZ_AFQT00000000.1
51	*Streptococcus mitis*	SK1080	NZ_AFQV00000000.1
52	*Streptococcus mitis*	SK1126	NZ_JPFT00000000.1
53	*Streptococcus mitis*	SK137	NZ_JPFS00000000.1
54	*Streptococcus mitis*	SK145	NZ_JYGS00000000.1
55	*Streptococcus mitis*	SK321	NZ_AEDT00000000.1
56	*Streptococcus mitis*	SK564	NZ_AEDU00000000.1
57	*Streptococcus mitis*	SK569	NZ_AFUF00000000.1
58	*Streptococcus mitis*	SK575	NZ_AICU00000000.1
59	*Streptococcus mitis*	SK578	NZ_JPFY00000000.1
60	*Streptococcus mitis*	SK579	NZ_AJJL00000000.1
61	*Streptococcus mitis*	SK597	NZ_AEDV00000000.1
62	*Streptococcus mitis*	SK608	NZ_JPFZ00000000.1
63	*Streptococcus mitis*	SK616	NZ_AICR00000000.1
64	*Streptococcus mitis*	SK629	NZ_JPFU00000000.1
65	*Streptococcus mitis*	SK637	NZ_JPFX00000000.1
66	*Streptococcus mitis*	SK642	NZ_JPFW00000000.1
67	*Streptococcus mitis*	SK667	NZ_JPFV00000000.1
68	*Streptococcus oralis*	1314_SORA	NZ_JVUD00000000.1
69	*Streptococcus oralis*	727_SORA	NZ_JUVM00000000.1
70	*Streptococcus oralis*	734_SORA	NZ_JUVF00000000.1
71	*Streptococcus oralis*	918_SORA	NZ_JUNW00000000.1
72	*Streptococcus oralis*	ATCC 35037T	NZ_ADMV00000000.1
73	*Streptococcus oralis*	ATCC 49296	NZ_AEPO00000000.1
74	*Streptococcus oralis*	SK10	NZ_AJKO00000000.1
75	*Streptococcus oralis*	SK100	NZ_AJKP00000000.1
76	*Streptococcus oralis*	SK141	NZ_JPGA00000000.1
77	*Streptococcus oralis*	SK143	NZ_JPGB00000000.1
78	*Streptococcus oralis*	SK610	NZ_AJKQ00000000.1
79	*Streptococcus oralis*	Uo5	NC_015291.1
80	*Streptococcus oralis*	AZ_3a	NZ_AORU00000000.1
81	*Streptococcus oralis*	1366	NZ_AORX00000000.1
82	*Streptococcus oralis*	2425	NZ_ASWZ00000000.1
83	*Streptococcus oralis*	2426	NZ_ASXA00000000.1
84	*Streptococcus oralis*	CCUG 35754	NZ_LWCE00000000.1
85	Streptococcus australis	ATCC 700641T	NZ_AEQR00000000.1
86	Streptococcus cristatus	ATCC 51100T	NZ_AEVC00000000.1
87	Streptococcus gordonii	CCUG 33482T	LQWV00000000.1
88	Streptococcus gordonii	KCOM 1506 (= ChDC B679)	NZ_CP012648.1
89	Streptococcus gordonii	CH1	NC_009785.1
90	Streptococcus infantis	ATCC 7007779T	NZ_AEVD00000000.1
91	Streptococcus oligofermentans	AS 1.3089T	NC_021175.1
92	Streptococcus parasanguinis	ATCC 15912T	NC_015678.1
93	Streptococcus parasanguinis	F0449	NZ_AJMV00000000.1
94	Streptococcus parasanguinis	FW213	NC_017905.1
95	Streptococcus peroris	ATCC 700780	NZ_AEVF00000000.1
96	Streptococcus sanguinis	CC94A	NZ_AZJC00000000.1
97	Streptococcus sanguinis	SK1058	NZ_AFBF00000000.1
98	Streptococcus sanguinis	SK36	NC_009009.1

99	Streptococcus sanguinis	SK405	NZ_AEWZ00000000.1
100	Streptococcus sanguinis	SK408	NZ_AFBE00000000.1
101	Streptococcus sanguinis	SK678	NZ_AEXA00000000.1
102	Streptococcus sinensis	HKU4T	NZ_JPEN00000000.1
