# Supplementary material for: Proteotyping bacteria: Characterization, differentiation and identification of pneumococcus and other species within the Mitis Group of the genus Streptococcus by tandem mass spectrometry proteomics
Source: PLoS One. 2018 Dec 10;13(12):e0208804. doi: 10.1371/journal.pone.0208804 (PMC6287849; doi:10.1371/journal.pone.0208804)
Supplement: S3 Table — (PDF) [file pone.0208804.s003.pdf]

S3 Table. Information about the twelve strains used as proteotyping model system

CLINICAL DATA

Strain	Organism	Isolation source	Place	Date of isolation
CCUG 4207T	*S. pyogenes*	Patient with scarlet fever	Manchester, UK	Before 1926
CCUG 25570	*S. pyogenes*	Human	Halmstad, Sweden	1985
CCUG 47803	*S. pyogenes*	Human infected wound	Unknown	Unknown
CCUG 49455T	*S. pseudopneumoniae*	Human sputum	Halifax, Canada	2002
CCUG 63747	*S. pseudopneumoniae*	Human	Boråas, Sweden	2013
CCUG 62647	*S. pseudopneumoniae*	Human pharynx	Aarhus, Denmark	2000
CCUG 31611T	*S. mitis*	Human oral cavity	Sweden	Unknown
CCUG 63687	*S. mitis*	Human blood	Jönköping, Sweden	2012
CCUG 69183 = SK271	*S. mitis*	Human mouth	Denmark	2014-10
CCUG 28588T	*S. pneumoniae*	Unknown	New York, NY, USA	1948
CCUG 7206	*S. pneumoniae*	Human blood	Gothenburg, Sweden	1978
CCUG 35180	*S. pneumoniae*	Human blood	Gothenburg, Sweden	1995

GENOMIC DATA

Strain	Organism	Accession no.	DNA extraction method	Illumina	Assembly
CCUG 4207T	*S. pyogenes*	MLGC00000000.1	Promega	HiSeq 2500	CLC Genomic Workbench 9.5.1
CCUG 25570	*S. pyogenes*	LNPL00000000.1	Promega	HiSeq 2500	CLC Genomic Workbench 8.0.1
CCUG 47803	*S. pyogenes*	MUYP00000000.1	Marmur procedure	MiSeq	CLC Genomic Workbench 9.5.1
CCUG 49455T	*S. pseudopneumoniae*	MWSM00000000.1	Magna Pure Compact	MiSeq	CLC Genomic Workbench 10.0
CCUG 63747	*S. pseudopneumoniae*	MUXR00000000.1	Magna Pure Compact	MiSeq	CLC Genomic Workbench 9.5.1
CCUG 62647	*S. pseudopneumoniae*	MUXQ00000000.1	Magna Pure Compact	MiSeq	CLC Genomic Workbench 9.5.1
CCUG 31611T	*S. mitis*	MUYN00000000.1	Magna Pure Compact	MiSeq	CLC Genomic Workbench 9.5.1
CCUG 63687	*S. mitis*	MUYO00000000.1	Magna Pure Compact	MiSeq	CLC Genomic Workbench 9.5.1
CCUG 69183 = SK271	*S. mitis*	JPGW00000000.1	PMID: 25053789	PMID: 25053789	PMID: 25053789
CCUG 28588T	*S. pneumoniae*	LQXF00000000.1	Promega	HiSeq 2500	CLC Genomic Workbench 8.0.1
CCUG 7206	*S. pneumoniae*	LWCD00000000.1	Magna Pure Compact	MiSeq	CLC Genomic Workbench 8.0.1
CCUG 35180	*S. pneumoniae*	LQQK00000000.1	Magna Pure Compact	MiSeq	CLC Genomic Workbench 8.0.1
