# Supplementary material for: Proteotyping bacteria: Characterization, differentiation and identification of pneumococcus and other species within the Mitis Group of the genus Streptococcus by tandem mass spectrometry proteomics
Source: PLoS One. 2018 Dec 10;13(12):e0208804. doi: 10.1371/journal.pone.0208804 (PMC6287849; doi:10.1371/journal.pone.0208804)
Supplement: S4 Table — (PDF) [file pone.0208804.s004.pdf]

S4 Table.

List of proteins identified by species-unique peptides in analysis of *S. pneumoniae* CCUG 28588T

Accession number	Description	Nº peptides	Coverage
WP_001035310.1	hypothetical protein	25	36,3
WP_000685088.1	UDP-glucose dehydrogenase	11	44,1
WP_001844726.1	endo-beta-N-acetylglucosaminidase	10	8,5
WP_065251743.1	choline-binding protein	10	23,5
WP_000679960.1	beta-N-acetylhexosaminidase	8	7,9
WP_001214397.1	NAD-dependent dehydratase	7	24,4
WP_000727933.1	foldase	6	24,3
WP_000434652.1	thiol reductase thioredoxin	4	44,3
WP_000495824.1	transcriptional regulator	4	42,6
WP_000811753.1	alanine--tRNA ligase	4	4,0
WP_000036661.1	dihydroxyacetone kinase	3	8,6
WP_000064115.1	general stress protein	3	23,8
WP_000767195.1	hypothetical protein	3	13,1
WP_000862350.1	glycosyl transferase family 1	3	10,4
WP_001079795.1	galacturonic acid acetylase	3	21,9
WP_001818788.1	PTS glucose transporter subunit IIABC	3	6,3
WP_000116461.1	trigger factor	2	8,4
WP_000164758.1	glycine--tRNA ligase subunit beta	2	4,0
WP_000201902.1	RNA polymerase sigma factor SigA	2	5,1
WP_000245505.1	30S ribosomal protein S8	2	14,4
WP_000411198.1	choline kinase	2	10,4
WP_000432756.1	alpha-mannosidase	2	5,4
WP_000529016.1	serine protease	2	4,3
WP_000599104.1	ribosome-associated factor Y	2	6,0
WP_000639574.1	hypothetical protein	2	42,9
WP_000664173.1	capsular polysaccharide biosynthesis protein CpsC	2	16,1
WP_000701442.1	PTS fructose transporter subunit IIC	2	5,4
WP_000790743.1	hypothetical protein	2	15,5
WP_001032504.1	YSIRK signal domain/LPXTG anchor domain surface protein	2	2,0
WP_001092741.1	arginine--tRNA ligase	2	5,5
WP_001162938.1	dihydrolipoyl dehydrogenase	2	5,3
WP_001229596.1	arginine ABC transporter ATP-binding protein	2	15,5
WP_001232820.1	alkaline amylopullulanase	2	3,8
WP_001818543.1	cell division protein DivIVA	2	11,1
WP_000003933.1	S-adenosylmethionine synthase	1	4,0
WP_000010163.1	ribose-phosphate pyrophosphokinase 1	1	3,4
WP_000018260.1	glucose-6-phosphate isomerase	1	4,0
WP_000022100.1	purine-nucleoside phosphorylase	1	5,9
WP_000029860.1	cysteine synthase A	1	7,8
WP_000036769.1	NADH oxidase	1	3,3
WP_000039205.1	translation initiation factor IF-2	1	2,8
WP_000039622.1	FMN reductase	1	5,5
WP_000053388.1	cell division protein SepF	1	15,6
WP_000081003.1	ferredoxin--NADP(+) reductase	1	4,0
WP_000086630.1	50S ribosomal protein L6	1	8,4
WP_000109138.1	50S ribosomal protein L21	1	10,6

WP_000109957.1	CsbD family protein	1	26,9
WP_000115730.1	MULTISPECIES: hypothetical protein [Streptococcus]	1	13,7
WP_000121713.1	phosphoglucomutase	1	2,1
WP_000136824.1	membrane protein	1	4,2
WP_000144280.1	cell division protein FtsZ	1	2,9
WP_000158725.1	isochorismatase	1	9,4
WP_000163684.1	methionyl-tRNA formyltransferase	1	5,1
WP_000173353.1	bacteriocin ABC transporter ATP-binding protein	1	6,0
WP_000179632.1	DNA topoisomerase I	1	2,3
WP_000196418.1	Fe-S cluster assembly protein SufD	1	6,7
WP_000221752.1	hydroxymethylpyrimidine/phosphomethylpyrimidine kinase	1	3,8
WP_000229878.1	DNA starvation/stationary phase protection protein	1	12,2
WP_000257841.1	acyl carrier protein	1	18,9
WP_000268677.1	CBS domain-containing protein	1	6,0
WP_000283061.1	pullulanase	1	2,4
WP_000334275.1	glutamine--fructose-6-phosphate aminotransferase	1	3,2
WP_000348122.1	aquaporin	1	5,5
WP_000380924.1	GTP cyclohydrolase I FolE	1	6,5
WP_000386347.1	MarR family transcriptional regulator	1	10,4
WP_000395924.1	alpha-glycerophosphate oxidase	1	1,5
WP_000404940.1	ribonuclease Y	1	2,4
WP_000417162.1	peptidase M26	1	0,6
WP_000453139.1	alanine aminotransferase	1	3,2
WP_000472986.1	zinc metalloprotease zmpB	1	1,0
WP_000510591.1	hypothetical protein	1	7,8
WP_000517372.1	hypothetical protein	1	14,0
WP_000542470.1	aspartate-semialdehyde dehydrogenase	1	3,6
WP_000546881.1	tyrosine--tRNA ligase	1	6,7
WP_000570244.1	DUF4649 domain-containing protein	1	16,4
WP_000584891.1	multidrug ABC transporter ATP-binding protein	1	2,8
WP_000624331.1	glutamine ABC transporter substrate-binding protein	1	7,1
WP_000634929.1	sialidase	1	1,9
WP_000649200.1	zinc-dependent alcohol dehydrogenase	1	4,7
WP_000666471.1	aminotransferase AlaT	1	2,7
WP_000671133.1	DEAD/DEAH box family ATP-dependent RNA helicase	1	2,9
WP_000686956.1	hypothetical protein	1	3,8
WP_000705433.1	aspartyl/glutamyl-tRNA(Asn/Gln) amidotransferase subunit C	1	11,0
WP_000724951.1	amino acid ABC transporter substrate-binding protein	1	4,0
WP_000729037.1	hypothetical protein	1	10,7
WP_000750087.1	serine protease	1	0,7
WP_000768069.1	isoleucine--tRNA ligase	1	1,4
WP_000777760.1	MULTISPECIES: hypothetical protein [Streptococcus]	1	13,7
WP_000797065.1	Fe-S cluster assembly protein SufB	1	2,3

WP_000805597.1	5-(carboxyamino)imidazole ribonucleotide mutase	1	8,0
WP_000814633.1	D-alanine--D-alanine ligase	1	3,7
WP_000825669.1	PEP phosphonomutase	1	4,0
WP_000845290.1	formate--tetrahydrofolate ligase	1	2,3
WP_000850024.1	bifunctional protein PyrR	1	9,2
WP_000856522.1	diaminopimelate decarboxylase	1	3,6
WP_000863038.1	UDP-N-acetylmuramoylalanine--D-glutamate ligase	1	3,6
WP_000887724.1	cysteine desulfurase	1	3,7
WP_000892184.1	hypoxanthine phosphoribosyltransferase	1	7,2
WP_000907145.1	MULTISPECIES: DNA-directed RNA polymerase subunit beta	1	1,2
WP_000940733.1	GTP-sensing transcriptional pleiotropic repressor CodY	1	5,7
WP_000990607.1	catabolite control protein A	1	4,2
WP_001034417.1	arginine repressor	1	9,1
WP_001042590.1	DNA ligase (NAD(+)) LigA	1	2,1
WP_001047204.1	elongation factor 4	1	2,1
WP_001063454.1	ATP-dependent Clp protease ATP-binding subunit	1	1,6
WP_001064551.1	PTS beta-glucoside transporter subunit EIIBCA	1	2,4
WP_001140347.1	hypothetical protein	1	4,5
WP_001140486.1	prephenate dehydrogenase	1	3,8
WP_001142516.1	tyrosine protein kinase	1	5,9
WP_001145974.1	cytosine-specific methyltransferase NlaX	1	3,0
WP_001162129.1	flavodoxin	1	7,5
WP_001262221.1	ribosome recycling factor	1	5,9
WP_001291372.1	methionine--tRNA ligase	1	2,7
WP_001291649.1	aminodeoxychorismate lyase	1	1,8
WP_001844622.1	carbamoyl phosphate synthase large subunit	1	1,4
WP_001844943.1	DNA-directed RNA polymerase subunit omega	1	18,3
