# Supplementary material for: Proteotyping bacteria: Characterization, differentiation and identification of pneumococcus and other species within the Mitis Group of the genus Streptococcus by tandem mass spectrometry proteomics
Source: PLoS One. 2018 Dec 10;13(12):e0208804. doi: 10.1371/journal.pone.0208804 (PMC6287849; doi:10.1371/journal.pone.0208804)
Supplement: S5 Table — (PDF) [file pone.0208804.s005.pdf]

S5 Table.
List of proteins identified by species-unique peptides in analysis of *S. pneumoniae* CCUG 7206

Accession number	Description	Nº peptides	Coverage
WP_063642986.1	sialidase	11	13,0
WP_000417171.1	immunoglobulin A1 protease	8	9,3
WP_000727935.1	foldase	6	15,7
WP_000064115.1	general stress protein	4	15,3
WP_000094618.1	arginine deiminase	4	11,7
WP_001162912.1	dihydrolipoamide dehydrogenase	4	12,0
WP_001855682.1	endo-beta-N-acetylglucosaminidase	4	2,6
WP_000186955.1	membrane protein	3	8,6
WP_000448317.1	N-acetylmannosamine-6-phosphate 2-epimerase	3	19,7
WP_000686980.1	hypothetical protein	3	9,7
WP_050273778.1	zinc metalloprotease	3	2,7
WP_063643216.1	hypothetical protein	3	5,4
WP_000105362.1	pyruvate dehydrogenase E1 subunit alpha	2	8,1
WP_000120709.1	lactate oxidase	2	4,0
WP_000167757.1	acetate kinase	2	8,6
WP_000185363.1	ornithine carbamoyltransferase	2	10,4
WP_000434650.1	thiol reductase thioredoxin	2	18,3
WP_000546887.1	tyrosine--tRNA ligase	2	9,8
WP_000671113.1	DEAD/DEAH box family ATP-dependent RNA helicase	2	5,5
WP_000790743.1	hypothetical protein	2	7,9
WP_001029637.1	PTS cellbiose transporter subunit IIC	2	15,4
WP_001085463.1	DNA recombination/repair protein RecA	2	4,9
WP_001092701.1	arginine--tRNA ligase	2	3,7
WP_001232811.1	alkaline amylopullulanase	2	2,7
WP_050146947.1	glycine--tRNA ligase subunit beta	2	4,0
WP_061694626.1	beta-N-acetylhexosaminidase	2	1,6
WP_063643199.1	trigger factor	2	7,5
WP_000003935.1	S-adenosylmethionine synthase	1	4,0
WP_000031585.1	molecular chaperone GroEL	1	3,3
WP_000033099.1	phosphopentomutase	1	3,5
WP_000039210.1	translation initiation factor IF-2	1	1,9
WP_000086630.1	50S ribosomal protein L6	1	8,4
WP_000095485.1	sugar ABC transporter substrate-binding protein	1	4,0
WP_000105270.1	GntR family transcriptional regulator	1	5,0
WP_000109957.1	CsbD family protein	1	16,4
WP_000121704.1	phosphoglucomutase	1	2,4
WP_000136830.1	membrane protein	1	3,9
WP_000141506.1	dTDP-glucose 4,6-dehydratase	1	4,3
WP_000144280.1	cell division protein FtsZ	1	2,9
WP_000201898.1	RNA polymerase sigma factor SigA	1	3,5
WP_000245505.1	30S ribosomal protein S8	1	10,6
WP_000257841.1	acyl carrier protein	1	18,9
WP_000260018.1	DNA-binding protein	1	6,4
WP_000268662.1	CBS domain-containing protein	1	6,0
WP_000348122.1	aquaporin	1	5,5
WP_000370269.1	glycogen-branching enzyme	1	2,2
WP_000404940.1	ribonuclease Y	1	2,4

WP_000510591.1	hypothetical protein	1	7,8
WP_000575212.1	glycine/betaine ABC transporter ATP-binding protein	1	7,0
WP_000600913.1	NAD(P)-dependent oxidoreductase	1	3,9
WP_000705418.1	glutamyl-tRNA amidotransferase subunit C	1	14,0
WP_000706826.1	N-acetylgalactosamine transporter subunit IIA	1	9,0
WP_000729030.1	hypothetical protein	1	10,7
WP_000742290.1	hypothetical protein	1	5,0
WP_000744557.1	ATP-dependent zinc metalloprotease FtsH	1	2,1
WP_000767193.1	hypothetical protein	1	12,1
WP_000850024.1	bifunctional protein PyrR	1	9,2
WP_000863023.1	UDP-N-acetylmuramoylalanine--D-glutamate ligase	1	3,6
WP_000887717.1	cysteine desulfurase	1	3,7
WP_000892185.1	hypoxanthine-guanine phosphoribosyltransferase	1	7,2
WP_000907135.1	DNA-directed RNA polymerase subunit beta	1	1,2
WP_000963678.1	DNA mismatch repair protein MutS	1	1,3
WP_000990607.1	catabolite control protein A	1	4,2
WP_000992853.1	ATP-dependent DNA helicase PcrA	1	2,2
WP_001032456.1	endo-alpha-N-acetylgalactosaminidase	1	0,6
WP_001034415.1	arginine repressor	1	9,1
WP_001047204.1	elongation factor 4	1	2,1
WP_001085888.1	aminoacyl-tRNA deacylase	1	6,9
WP_001096142.1	hypothetical protein	1	6,5
WP_001162129.1	flavodoxin	1	7,5
WP_001291409.1	methionine--tRNA ligase	1	2,7
WP_001291650.1	aminodeoxychorismate lyase	1	1,8
WP_001809950.1	50S ribosomal protein L33	1	18,0
WP_001818381.1	PTS glucose transporter subunit IIABC	1	2,5
WP_001818543.1	cell division protein DivIVA	1	5,0
WP_033705237.1	pullulanase	1	2,4
WP_050084726.1	NADPH-dependent FMN reductase	1	5,5
WP_050213965.1	Clp protease ClpX	1	1,4
WP_050256225.1	phosphorylcholine transferase LicD	1	6,3
WP_057485901.1	valine--tRNA ligase	1	1,4
WP_061372758.1	glutamate--tRNA ligase	1	3,3
WP_063642991.1	PTS beta-glucoside transporter subunit EIIBCA	1	2,4
WP_063643115.1	choline kinase	1	3,8
WP_063643118.1	endonuclease	1	2,0
WP_063643127.1	chromosome segregation protein SMC	1	1,2
WP_063643275.1	alanine aminotransferase	1	3,2
WP_063643338.1	glycerol kinase	1	3,6
WP_063643343.1	N-acetyl-beta-D-glucosaminidase	1	3,2
