# Supplementary material for: Proteotyping bacteria: Characterization, differentiation and identification of pneumococcus and other species within the Mitis Group of the genus Streptococcus by tandem mass spectrometry proteomics
Source: PLoS One. 2018 Dec 10;13(12):e0208804. doi: 10.1371/journal.pone.0208804 (PMC6287849; doi:10.1371/journal.pone.0208804)
Supplement: S6 Table — (PDF) [file pone.0208804.s006.pdf]

S6 Table.

List of proteins identified by species-unique peptides in analysis of *S. pneumoniae* CCUG 35180

Accession number	Description	Nº peptides	Coverage
WP_054381254.1	choline-binding protein	25	33,5
WP_000634963.1	sialidase	19	27,0
WP_000064115.1	general stress protein	5	23,8
WP_000863391.1	sugar-binding protein	5	22,6
WP_000094617.1	arginine deiminase	4	11,7
WP_000686975.1	hypothetical protein	4	14,2
WP_000727935.1	foldase	4	15,3
WP_001032494.1	endo-alpha-N-acetylgalactosaminidase	4	3,3
WP_001162695.1	dihydrolipoyl dehydrogenase	4	12,0
WP_001232790.1	alkaline amylopullulanase	4	3,3
WP_001291665.1	aminodeoxychorismate lyase	4	8,5
WP_000053388.1	cell division protein SepF	3	16,2
WP_000121704.1	phosphoglucomutase	3	7,9
WP_000434644.1	thiol reductase thioredoxin	3	28,7
WP_000599104.1	ribosome-associated factor Y	3	8,8
WP_000767195.1	hypothetical protein	3	13,1
WP_000790743.1	hypothetical protein	3	12,6
WP_000837080.1	cell wall-binding protein	3	7,1
WP_000880678.1	tail protein	3	17,5
WP_001820922.1	endo-beta-N-acetylglucosaminidase	3	2,3
WP_000011015.1	DEAD/DEAH box helicase	2	5,8
WP_000018263.1	glucose-6-phosphate isomerase	2	6,9
WP_000036674.1	dihydroxyacetone kinase	2	6,3
WP_000086630.1	50S ribosomal protein L6	2	8,4
WP_000116465.1	trigger factor	2	7,5
WP_000164772.1	glycine--tRNA ligase subunit beta	2	4,0
WP_000185363.1	ornithine carbamoyltransferase	2	10,4
WP_000395959.1	alpha-glycerophosphate oxidase	2	4,8
WP_000515192.1	beta-galactosidase	2	1,9
WP_000679942.1	beta-N-acetylhexosaminidase	2	2,1
WP_000723325.1	UDP-N-acetyl glucosamine 2-epimerase	2	8,8
WP_001196960.1	50S ribosomal protein L7/L12	2	15,6
WP_041172731.1	peptidase M26, partial	2	1,9
WP_050200722.1	peptidoglycan GlcNAc deacetylase	2	6,0
WP_000003936.1	S-adenosylmethionine synthase	1	4,0
WP_000010157.1	ribose-phosphate pyrophosphokinase	1	3,4
WP_000011758.1	leucine--tRNA ligase	1	1,9
WP_000013420.1	carbamate kinase	1	4,8
WP_000021975.1	MULTISPECIES: PTS mannose transporter subunit EIIAB	1	4,0
WP_000022102.1	purine-nucleoside phosphorylase	1	5,9
WP_000031090.1	glutamate--tRNA ligase	1	3,3
WP_000032242.1	valine--tRNA ligase	1	1,4
WP_000041492.1	addiction module antitoxin RelB	1	13,8
WP_000081011.1	ferredoxin--NADP(+) reductase	1	4,0
WP_000083614.1	hypothetical protein	1	5,9
WP_000090344.1	elongation factor G	1	1,7

WP_000120709.1	lactate oxidase	1	4,0
WP_000136830.1	membrane protein	1	3,9
WP_000144280.1	cell division protein FtsZ	1	2,9
WP_000167622.1	malonyl CoA-acyl carrier protein transacylase	1	4,6
WP_000167766.1	acetate kinase	1	4,5
WP_000201898.1	RNA polymerase sigma factor SigA	1	3,5
WP_000202235.1	UTP--glucose-1-phosphate uridylyltransferase	1	4,0
WP_000221749.1	hydroxymethylpyrimidine/phosphomethylpyrimidine kinase	1	3,8
WP_000245505.1	30S ribosomal protein S8	1	10,6
WP_000257841.1	acyl carrier protein	1	18,9
WP_000260025.1	DNA-binding protein	1	6,4
WP_000268677.1	CBS domain-containing protein	1	6,0
WP_000280960.1	chromosome segregation protein SMC	1	1,2
WP_000283118.1	nicotinate phosphoribosyltransferase	1	2,5
WP_000334265.1	glutamine--fructose-6-phosphate aminotransferase	1	3,0
WP_000348126.1	aquaporin	1	5,5
WP_000354338.1	ribonuclease Z	1	4,5
WP_000359340.1	PTS lactose transporter subunit IIBC	1	2,0
WP_000386347.1	MarR family transcriptional regulator	1	10,4
WP_000404940.1	ribonuclease Y	1	2,4
WP_000453143.1	alanine aminotransferase	1	3,2
WP_000473015.1	zinc metalloprotease	1	0,5
WP_000477450.1	PTS glucose transporter subunit IIBC	1	3,4
WP_000517372.1	hypothetical protein	1	14,0
WP_000522316.1	signal recognition particle-docking protein FtsY	1	6,8
WP_000569207.1	3-dehydroquinate dehydratase	1	4,4
WP_000570244.1	DUF4649 domain-containing protein	1	16,4
WP_000590986.1	amino acid ABC transporter ATP-binding protein	1	6,1
WP_000676130.1	glucose-1-phosphate thymidylyltransferase	1	5,2
WP_000728634.1	transporter	1	4,3
WP_000744551.1	cell division protein FtsH	1	2,1
WP_000797073.1	Fe-S cluster assembly protein SufB	1	2,3
WP_000808456.1	membrane protein	1	4,0
WP_000809624.1	PTS sugar transporter subunit IIB	1	13,7
WP_000811723.1	alanine--tRNA ligase	1	1,0
WP_000830868.1	aspartate--tRNA ligase	1	1,9
WP_000850024.1	bifunctional protein PyrR	1	9,2
WP_000852249.1	hypothetical protein	1	12,9
WP_000863026.1	UDP-N-acetylmuramoylalanine--D-glutamate ligase	1	3,6
WP_000882537.1	ATP-dependent Clp protease ATP-binding subunit	1	1,6
WP_000884255.1	serine--tRNA ligase	1	3,5
WP_000887726.1	cysteine desulfurase	1	3,7
WP_000892185.1	hypoxanthine-guanine phosphoribosyltransferase	1	7,2
WP_000903552.1	tagatose-6-phosphate ketose	1	3,9
WP_000907135.1	DNA-directed RNA polymerase subunit beta	1	1,2
WP_000940733.1	GTP-sensing transcriptional pleiotropic repressor CodY	1	5,7

WP_000958925.1	pyridine nucleotide-disulfide oxidoreductase	1	3,0
WP_000963680.1	DNA mismatch repair protein MutS	1	1,3
WP_000990607.1	catabolite control protein A	1	4,2
WP_000991679.1	aminopeptidase C	1	3,4
WP_000992885.1	ATP-dependent DNA helicase PcrA	1	2,2
WP_001034412.1	arginine repressor	1	9,1
WP_001042568.1	DNA ligase (NAD(+)) LigA	1	2,1
WP_001047220.1	elongation factor 4	1	2,0
WP_001061594.1	hypothetical protein	1	9,8
WP_001077246.1	peptidase M42	1	3,2
WP_001085463.1	DNA recombination/repair protein RecA	1	4,4
WP_001099660.1	2,5-diketo-D-gluconic acid reductase	1	4,3
WP_001156827.1	glucan 1,6-alpha-glucosidase	1	3,6
WP_001157038.1	hypothetical protein	1	3,8
WP_001199649.1	phosphorylcholine transferase LicD	1	6,3
WP_001200062.1	tRNA sulfurtransferase ThiI	1	3,0
WP_001212028.1	Xaa-Pro dipeptidyl-peptidase	1	1,6
WP_001281489.1	N-acetylneuraminate lyase	1	5,6
WP_001284361.1	pneumolysin	1	2,8
WP_001291369.1	methionine--tRNA ligase	1	2,7
WP_001810114.1	cell division protein DivIVA	1	5,0
WP_001818381.1	PTS glucose transporter subunit IIABC	1	2,5
WP_054409198.1	translation initiation factor IF-2	1	1,9
WP_054409214.1	aminotransferase V	1	3,7
WP_054409227.1	flavodoxin	1	7,5
WP_063612813.1	tyrosine protein kinase	1	5,7
WP_063612814.1	capsular biosynthesis protein CpsC	1	4,8
WP_063612827.1	hypothetical protein, partial	1	3,8
