# Supplementary material for: Proteotyping bacteria: Characterization, differentiation and identification of pneumococcus and other species within the Mitis Group of the genus Streptococcus by tandem mass spectrometry proteomics
Source: PLoS One. 2018 Dec 10;13(12):e0208804. doi: 10.1371/journal.pone.0208804 (PMC6287849; doi:10.1371/journal.pone.0208804)
Supplement: S7 Table — (PDF) [file pone.0208804.s007.pdf]

S7 Table.

List of proteins identified by species-unique peptides in analysis of *S. mitis* CCUG 31611T

Accession number	Description	Nº peptides	Coverage
OOS15439.1	choline-binding protein A	22	41,2
OOS15436.1	maebl	10	11,2
OOS16375.1	YSIRK signal domain/LPXTG anchor domain surface protein	8	4,1
OOS17165.1	pullulanase	8	9,2
OOS15437.1	PspC family transcriptional regulator	6	18,8
OOS15639.1	septation ring formation regulator EzrA	6	13,2
OOS16702.1	cell division protein FtsZ	6	22,0
OOS15438.1	nucleoside triphosphate hydrolase	5	14,3
OOS15642.1	ribose-5-phosphate isomerase	5	30,3
OOS15740.1	foldase PrsA	5	17,9
OOS16219.1	peptide ABC transporter ATP-binding protein	5	10,7
OOS16700.1	cell division protein SepF	5	37,3
OOS16703.1	cell division protein FtsA	5	14,6
OOS17049.1	hypothetical protein B0679_00520	5	21,8
OOS15407.1	GntR family transcriptional regulator	4	13,4
OOS16697.1	cell division protein DivIVA	4	25,8
OOS16994.1	ribosomal subunit interface protein	4	12,6
OOS17342.1	DNA-directed RNA polymerase subunit omega	4	18,3
OOS14650.1	protein jag	3	11,6
OOS15287.1	zinc metalloprotease	3	2,6
OOS15399.1	choline-binding protein C	3	6,3
OOS15897.1	thiol reductase thioredoxin	3	27,0
OOS16247.1	peptide ABC transporter ATP-binding protein	3	7,3
OOS16777.1	general stress protein	3	27,7
OOS17016.1	alpha-glycerophosphate oxidase	3	3,5
OOS17172.1	recombinase RecA	3	8,6
OOS14652.1	transketolase	2	4,1
OOS14902.1	CBS domain-containing protein	2	11,5
OOS14906.1	PTS glucose/maltose transporter subunit IIBCA	2	4,5
OOS15272.1	aminodeoxychorismate lyase	2	5,3
OOS15388.1	potassium transporter Trk	2	13,1
OOS15405.1	PTS mannose transporter subunit IIAB	2	8,9
OOS15620.1	efflux transporter periplasmic adaptor subunit	2	16,0
OOS15742.1	phosphoenolpyruvate carboxylase	2	3,0
OOS15963.1	phosphorylcholine transferase LicD	2	9,7
OOS16003.1	lysine--tRNA ligase	2	4,6
OOS16057.1	NAD(P)H-dependent oxidoreductase	2	10,4
OOS16060.1	glycine--tRNA ligase subunit beta	2	6,6
OOS16204.1	PTS mannose transporter subunit EIIAB	2	11,2
OOS16401.1	hypothetical protein B0679_04510	2	5,0
OOS16718.1	sugar ABC transporter substrate-binding protein	2	9,3
OOS16964.1	serine protease	2	7,4
OOS16986.1	CHAP domain-containing protein	2	5,1
OOS17207.1	phenylalanine--tRNA ligase subunit beta	2	3,7
OOS17209.1	sodium ABC transporter permease	2	9,5
OOS17305.1	enoyl-CoA hydratase	2	9,2
OOS17306.1	MarR family transcriptional regulator	2	16,7

OOS17307.1	ketoacyl-ACP synthase III	2	14,2
OOS17311.1	beta-ketoacyl-ACP reductase	2	6,6
OOS17346.1	protein phosphatase	2	11,4
OOS17369.1	polyribonucleotide nucleotidyltransferase	2	2,4
OOS14630.1	peptidoglycan-binding protein LysM	1	5,2
OOS14647.1	acetate kinase	1	4,0
OOS14905.1	cell division protein FtsX	1	7,8
OOS14908.1	DEAD/DEAH box helicase	1	2,0
OOS15245.1	aspartate aminotransferase	1	3,6
OOS15269.1	UDP-N-acetylmuramate--L-alanine ligase	1	2,7
OOS15288.1	matrix-binding protein, partial	1	1,9
OOS15393.1	muramidase	1	0,8
OOS15428.1	choline-binding protein C	1	3,0
OOS15615.1	hypothetical protein B0679_05130	1	11,6
OOS15622.1	hypothetical protein B0679_05165	1	2,6
OOS15636.1	DJ-1 family protein	1	7,6
OOS15652.1	ribose-5-phosphate isomerase	1	9,3
OOS15674.1	peptide ABC transporter ATP-binding protein	1	2,3
OOS15681.1	cysteine desulfurase	1	3,2
OOS15684.1	D-alanyl-D-alanine carboxypeptidase	1	3,9
OOS15699.1	gamma-glutamyl-phosphate reductase	1	2,9
OOS15700.1	pyrroline-5-carboxylate reductase	1	3,8
OOS15754.1	ATP-dependent 6-phosphofructokinase	1	5,4
OOS15770.1	arginine repressor	1	9,1
OOS15780.1	tagatose-6-phosphate kinase	1	4,5
OOS15806.1	pyruvate dehydrogenase	1	6,5
OOS15809.1	dihydrolipoyl dehydrogenase	1	1,9
OOS15848.1	hypothetical protein B0679_06385	1	5,2
OOS15851.1	serine hydroxymethyltransferase	1	3,1
OOS15911.1	cysteine desulfurase	1	4,6
OOS15936.1	glucose-6-phosphate dehydrogenase	1	3,2
OOS15937.1	signal recognition particle-docking protein FtsY	1	3,3
OOS15990.1	DHH family phosphoesterase	1	4,8
OOS15992.1	nucleoid-associated protein, YbaB/EbfC family	1	15,2
OOS16041.1	metallophosphatase	1	5,4
OOS16055.1	NADH oxidase	1	2,4
OOS16058.1	NAD(P)H-dependent oxidoreductase	1	2,9
OOS16062.1	aldehyde-activating protein	1	13,0
OOS16063.1	2,5-diketo-D-gluconic acid reductase	1	5,7
OOS16197.1	aminopeptidase	1	4,3
OOS16213.1	catabolite control protein A	1	4,2
OOS16248.1	peptide ABC transporter permease	1	3,4
OOS16298.1	translation initiation factor IF-2	1	2,0
OOS16300.1	DNA-binding protein	1	10,3
OOS16320.1	hypothetical protein B0679_04085	1	13,7
OOS16380.1	1,4-beta-N-acetylmuramidase	1	2,4
OOS16390.1	ferredoxin--NADP(+) reductase	1	5,3
OOS16564.1	chlorohydrolase	1	3,8
OOS16584.1	3-dehydroquinase	1	5,3
OOS16604.1	phosphate transport system regulatory protein PhoU	1	4,6

OOS16617.1	diacylglyceryl transferase	1	14,0
OOS16620.1	HPr kinase/phosphorylase	1	5,4
OOS16629.1	beta-galactosidase	1	0,7
OOS16646.1	peptidase M24 family protein	1	3,9
OOS16667.1	antibiotic ABC transporter permease	1	3,9
OOS16687.1	endopeptidase	1	2,5
OOS16706.1	D-alanine--D-alanine ligase A	1	3,7
OOS16719.1	PTS glucose transporter subunit IIBC	1	3,4
OOS16721.1	acetylxylan esterase	1	3,4
OOS16727.1	3-deoxy-7-phosphoheptulonate synthase	1	3,2
OOS16728.1	preprotein translocase subunit SecA	1	2,4
OOS16752.1	acetoin reductase	1	5,1
OOS16793.1	DUF421 domain-containing protein	1	5,7
OOS16952.1	hypoxanthine phosphoribosyltransferase	1	9,4
OOS16973.1	tryptophan--tRNA ligase	1	7,3
OOS16988.1	translation elongation factor Ts	1	4,6
OOS16996.1	50S ribosomal protein L9	1	10,0
OOS17026.1	dTDP-glucose 4,6-dehydratase	1	3,9
OOS17047.1	hypothetical protein B0679_00510	1	4,9
OOS17083.1	ABC transporter permease	1	11,9
OOS17151.1	leucine--tRNA ligase	1	1,4
OOS17161.1	proline--tRNA ligase	1	2,9
OOS17171.1	competence/damage-inducible protein A	1	3,8
OOS17183.1	hypothetical protein B0679_01225	1	3,1
OOS17208.1	sodium ABC transporter ATP-binding protein	1	5,4
OOS17255.1	alpha-glucosidase	1	2,8
OOS17260.1	tyrosine protein kinase	1	5,6
OOS17270.1	capsular biosynthesis protein	1	3,6
OOS17271.1	galactofuranosyltransferase	1	4,5
OOS17281.1	Holliday junction resolvase	1	5,5
OOS17309.1	2-nitropropane dioxygenase	1	3,4
OOS17310.1	malonyl CoA-acyl carrier protein transacylase	1	5,2
OOS17315.1	acetyl-CoA carboxylase biotin carboxylase subunit	1	3,1
OOS17316.1	acetyl-CoA carboxylase subunit beta	1	3,8
OOS17317.1	acetyl-CoA carboxylase carboxyl transferase subunit alpha	1	10,6
OOS17324.1	aspartyl/glutamyl-tRNA amidotransferase subunit A	1	3,1
OOS17337.1	ribosome silencing factor RsfS	1	8,5
OOS17341.1	guanylate kinase	1	8,2
OOS17383.1	phenylalanine--tRNA ligase subunit alpha	1	3,4
