# Supplementary material for: Proteotyping bacteria: Characterization, differentiation and identification of pneumococcus and other species within the Mitis Group of the genus Streptococcus by tandem mass spectrometry proteomics
Source: PLoS One. 2018 Dec 10;13(12):e0208804. doi: 10.1371/journal.pone.0208804 (PMC6287849; doi:10.1371/journal.pone.0208804)
Supplement: S8 Table — (PDF) [file pone.0208804.s008.pdf]

S8 Table.

List of proteins identified by species-unique peptides in analysis of *S. mitis* CCUG 63687

Accession number	Description	Nº peptides	Coverage
OOS16424.1	choline-binding protein A	15	47,7
OOS19177.1	hypothetical protein B0686_04340	14	24,8
OOS16425.1	nucleoside triphosphate hydrolase	10	25,7
OOS17710.1	YSIRK signal domain/LPXTG anchor domain surface protein, partial	9	7,0
OOS18831.1	hypothetical protein B0686_02450	9	30,9
OOS16475.1	muramidase	8	5,8
OOS18546.1	hypothetical protein B0686_00900	8	12,6
OOS18691.1	glucose-1-phosphate adenylyltransferase subunit GlgD	8	18,7
OOS17472.1	phosphatidylethanolamine-binding protein	7	26,9
OOS17915.1	pullulanase	7	9,6
OOS19245.1	cell division protein FtsZ	7	18,7
OOS17800.1	choline-binding protein C	6	16,9
OOS18097.1	ribosome-associated protein	6	12,6
OOS18799.1	phosphatidylethanolamine-binding protein	6	52,4
OOS17619.1	YSIRK signal domain/LPXTG anchor domain surface protein	5	3,3
OOS18557.1	peptide ABC transporter ATP-binding protein	5	9,5
OOS19029.1	septation ring formation regulator EzrA	5	12,5
OOS18412.1	aminodeoxychorismate lyase	4	10,5
OOS18432.1	glucokinase	4	8,2
OOS18802.1	hypothetical protein B0686_02295	4	20,9
OOS18900.1	ATP-dependent 6-phosphofructokinase	4	10,1
OOS18916.1	foldase PrsA	4	14,1
OOS19246.1	cell division protein FtsA	4	13,5
OOS16426.1	PspC family transcriptional regulator	3	6,4
OOS16837.1	peptidoglycan-binding protein LysM	3	13,5
OOS17467.1	peptide ABC transporter ATP-binding protein	3	7,7
OOS17906.1	recombinase RecA	3	8,6
OOS18104.1	CHAP domain-containing protein	3	10,6
OOS19027.1	ribose-5-phosphate isomerase	3	26,3
OOS19047.1	hypothetical protein B0686_03595	3	9,5
OOS19049.1	efflux transporter periplasmic adaptor subunit	3	16,0
OOS19312.1	general stress protein	3	18,8
OOS16470.1	choline-binding protein C	2	5,5
OOS16549.1	GntR family transcriptional regulator	2	6,7
OOS16883.1	coat protein	2	10,6
OOS16918.1	protein jag	2	4,3
OOS17413.1	hydrolase	2	12,6
OOS17415.1	PTS mannose family transporter subunit IID	2	8,6
OOS17417.1	PTS mannose transporter subunit IIAB	2	9,3
OOS17433.1	peptide ABC transporter ATP-binding protein	2	4,6
OOS17576.1	choline-binding protein A	2	8,9
OOS17671.1	serine/threonine protein kinase	2	6,2
OOS17672.1	protein phosphatase	2	11,4
OOS17772.1	[acyl-carrier-protein] S-malonyltransferase	2	10,1
OOS17813.1	cell division protein GpsB	2	34,5
OOS17869.1	sodium ABC transporter permease	2	6,5

OOS18428.1	zinc metalloprotease	2	1,8
OOS18486.1	acetoin utilization protein	2	11,5
OOS18502.1	NADPH-dependent FMN reductase	2	10,4
OOS18563.1	hypothetical protein B0686_00985	2	0,8
OOS18801.1	hypothetical protein B0686_02290	2	11,1
OOS18823.1	bacteriocin ABC transporter ATP-binding protein	2	6,2
OOS18898.1	L-lactate dehydrogenase	2	5,2
OOS18951.1	alanine dehydrogenase	2	8,4
OOS19240.1	cell division protein DivIVA	2	10,0
OOS19243.1	cell division protein SepF	2	19,8
OOS19261.1	sugar ABC transporter substrate-binding protein	2	4,1
OOS19298.1	acetoin reductase	2	9,1
OOS16266.1	NADPH-dependent FMN reductase	1	7,1
OOS16434.1	choline-binding protein C	1	3,6
OOS16482.1	potassium transporter Trk	1	8,1
OOS16511.1	aspartate--tRNA ligase	1	2,2
OOS16518.1	sugar ABC transporter substrate-binding protein	1	3,8
OOS16528.1	2,3,4,5-tetrahydropyridine-2,6-dicarboxylate N-acetyltransferase	1	7,3
OOS16535.1	arginine--tRNA ligase	1	1,8
OOS16890.1	hypothetical protein B0686_09475	1	8,2
OOS16915.1	acetate kinase	1	5,1
OOS16921.1	L-ribulose-5-phosphate 4-epimerase	1	5,5
OOS16944.1	B-cell receptor associated protein-related protein	1	7,1
OOS17426.1	catabolite control protein A	1	4,2
OOS17435.1	hydrolase TatD	1	6,6
OOS17437.1	hypothetical protein B0686_08390	1	14,1
OOS17444.1	pur operon repressor	1	5,8
OOS17468.1	peptide ABC transporter permease	1	3,4
OOS17480.1	non-canonical purine NTP pyrophosphatase	1	5,0
OOS17493.1	endo-beta-N-acetylglucosaminidase	1	1,1
OOS17648.1	polyribonucleotide nucleotidyltransferase	1	1,2
OOS17696.1	accessory Sec system glycosyltransferase GtfA	1	3,8
OOS17761.1	membrane protease	1	4,8
OOS17776.1	MarR family transcriptional regulator	1	7,6
OOS17777.1	enoyl-CoA hydratase	1	5,7
OOS17789.1	molecular chaperone DnaJ	1	5,3
OOS17812.1	RNA methyltransferase	1	3,9
OOS17817.1	peptide ABC transporter ATP-binding protein	1	2,1
OOS17823.1	capsular biosynthesis protein	1	3,6
OOS17828.1	hypothetical protein B0686_05730	1	3,7
OOS17833.1	tyrosine protein kinase	1	5,6
OOS17834.1	capsular biosynthesis protein CpsC	1	9,1
OOS17837.1	peptide ABC transporter ATP-binding protein	1	2,3
OOS17896.1	hypothetical protein B0686_06090	1	2,9
OOS17907.1	competence/damage-inducible protein A	1	3,6
OOS17919.1	proline--tRNA ligase	1	2,8
OOS17971.1	GNAT family acetyltransferase	1	8,3
OOS17974.1	damage-inducible protein CinA	1	3,1
OOS17997.1	ABC transporter permease	1	10,9

OOS18017.1	tRNA (adenosine(37)-N6)-threonylcarbamoyltransferase complex dimerization subunit type 1 TsaB	1	4,8
OOS18041.1	hypothetical protein B0686_06875	1	3,7
OOS18069.1	glycerol kinase	1	3,4
OOS18096.1	DHH family phosphoesterase	1	2,1
OOS18100.1	YigZ family protein	1	6,2
OOS18106.1	rod shape-determining protein MreC	1	5,5
OOS18128.1	chromosomal replication initiation protein DnaA	1	2,6
OOS18139.1	cell division protein FtsH	1	2,6
OOS18389.1	mucus-binding protein	1	0,9
OOS18400.1	CsbD family protein	1	23,8
OOS18413.1	transcription elongation factor GreA	1	11,9
OOS18431.1	calcium-binding protein	1	8,3
OOS18442.1	DNA-binding response regulator	1	4,5
OOS18490.1	cell division protein FtsX	1	7,8
OOS18491.1	PTS glucose/maltose transporter subunit IIBCA	1	2,5
OOS18499.1	glycine--tRNA ligase subunit beta	1	2,4
OOS18519.1	haloacid dehalogenase	1	6,2
OOS18541.1	proteinase	1	5,0
OOS18545.1	GMP synthetase	1	1,9
OOS18551.1	F0F1 ATP synthase subunit delta	1	6,2
OOS18566.1	aminoacyl-tRNA deacylase	1	6,9
OOS18619.1	bifunctional pyr operon transcriptional regulator/uracil phosphoribosyltransferase	1	8,7
OOS18621.1	carbamoyl phosphate synthase small subunit	1	3,6
OOS18623.1	phosphorylcholine transferase LicD	1	6,3
OOS18642.1	endonuclease	1	2,4
OOS18652.1	amino acid ABC transporter permease	1	2,6
OOS18673.1	adenylate cyclase	1	6,9
OOS18682.1	thiol reductase thioredoxin	1	16,5
OOS18688.1	type I pullulanase	1	1,4
OOS18689.1	1,4-alpha-glucan branching enzyme	1	3,6
OOS18694.1	glycerate kinase	1	3,5
OOS18706.1	thioredoxin	1	7,4
OOS18713.1	mechanosensitive ion channel protein MscL	1	17,5
OOS18805.1	hypothetical protein B0686_02310	1	21,4
OOS18820.1	LytTR family transcriptional regulator	1	10,0
OOS18822.1	ATPase	1	3,2
OOS18830.1	bacteriocin immunity protein	1	10,1
OOS18852.1	dihydroxyacetone kinase subunit DhaK	1	4,3
OOS18870.1	galactose-6-phosphate isomerase	1	9,2
OOS18961.1	dTMP kinase	1	7,1
OOS18977.1	D-alanyl-D-alanine carboxypeptidase	1	3,9
OOS18988.1	peptide ABC transporter ATP-binding protein	1	2,3
OOS19011.1	NAD-dependent protein deacylase	1	6,6
OOS19017.1	ribose 5-phosphate isomerase A	1	4,4
OOS19025.1	ATP-dependent Clp protease ATP-binding subunit	1	3,7
OOS19032.1	DJ-1 family protein	1	9,2
OOS19093.1	adaptor protein MecA	1	5,7
OOS19105.1	hypothetical protein B0686_03925	1	6,3

OOS19106.1	hypothetical protein B0686_03930	1	9,8
OOS19134.1	phosphate-binding protein	1	4,5
OOS19142.1	diacylglyceryl transferase	1	14,0
OOS19192.1	30S ribosomal protein S15	1	10,4
OOS19244.1	YggS family pyridoxal phosphate enzyme	1	5,4
OOS19249.1	D-alanine--D-alanine ligase A	1	2,9
OOS19255.1	hypothetical protein B0686_04775	1	5,4
OOS19268.1	acetylxylan esterase	1	3,7
OOS19273.1	alanine racemase	1	4,1
OOS19276.1	3-deoxy-7-phosphoheptulonate synthase	1	3,2
OOS19277.1	preprotein translocase subunit SecA	1	2,4
OOS19341.1	MarR family transcriptional regulator	1	8,2
OOS19373.1	acyl-[acyl-carrier-protein] thioesterase	1	4,1
OOS19375.1	hypothetical protein B0686_04205	1	6,3
