# Supplementary material for: Proteotyping bacteria: Characterization, differentiation and identification of pneumococcus and other species within the Mitis Group of the genus Streptococcus by tandem mass spectrometry proteomics
Source: PLoS One. 2018 Dec 10;13(12):e0208804. doi: 10.1371/journal.pone.0208804 (PMC6287849; doi:10.1371/journal.pone.0208804)
Supplement: S9 Table — (PDF) [file pone.0208804.s009.pdf]

S9 Table.

List of proteins identified by species-unique peptides in analysis of *S. mitis* CCUG 69183 (SK271)

Accession number	Description	Nº peptides	Coverage
KER08226.1	LPXTG-motif cell wall anchor domain protein	54	41,3
KER08229.1	pneumococcal surface protein A	22	38,2
KER07351.1	LPXTG-motif cell wall anchor domain protein	15	8,1
KER08215.1	cell wall binding repeat family protein	10	23,1
KER07353.1	LPXTG-motif cell wall anchor domain protein	9	3,4
KER08228.1	pneumococcal surface protein A	9	24,2
KER07595.1	putative pullulanase	8	8,9
KER08664.1	LPXTG-motif cell wall anchor domain protein	8	2,7
KER07313.1	LPXTG-motif cell wall anchor domain protein	7	6,0
KER08350.1	cell division protein FtsZ	6	21,3
KER07242.1	LPXTG-motif cell wall anchor domain protein	5	7,4
KER07776.1	hypothetical protein SK271_1842	5	12,6
KER08424.1	lysM domain protein	5	6,8
KER08647.1	PPIC-type PPIASE domain protein	5	18,9
KER07318.1	LPXTG-motif cell wall anchor domain protein	4	1,9
KER07352.1	LPXTG-motif cell wall anchor domain protein	4	3,0
KER08227.1	cell wall binding repeat family protein	4	7,9
KER07719.1	putative membrane protein	3	11,6
KER07941.1	response regulator	3	6,8
KER08055.1	oligopeptide-binding protein sarA	3	5,8
KER08352.1	hypothetical protein SK271_0393	3	20,9
KER08355.1	divIVA domain protein	3	21,0
KER08548.1	septation ring formation regulator, EzrA family protein	3	6,4
KER08946.1	R3H domain protein	3	8,8
KER07312.1	LPXTG-motif cell wall anchor domain protein	2	1,8
KER07363.1	putative NAD(P)H nitroreductase	2	6,0
KER07412.1	DNA-directed RNA polymerase, omega subunit	2	14,4
KER07547.1	ABC-2 transporter family protein	2	6,6
KER07676.1	succinyl-diaminopimelate desuccinylase	2	10,7
KER07784.1	CHAP domain protein	2	5,2
KER08106.1	glycine--tRNA ligase, beta subunit	2	4,6
KER08120.1	CBS domain protein	2	11,5
KER08125.1	receptor ligand binding region family protein	2	6,0
KER08182.1	cysteine-rich secretory family protein	2	5,5
KER08275.1	response regulator	2	14,4
KER08298.1	diacetyl reductase	2	5,1
KER08349.1	cell division protein FtsA	2	9,8
KER08550.1	hypothetical protein SK271_0593	2	13,1
KER08815.1	oligopeptide-binding protein AmiA	2	3,9
KER08845.1	endo-beta-N-acetylglucosaminidase D	2	1,3
KER07216.1	maltose/maltodextrin-binding protein	1	3,8
KER07244.1	trkA-N domain protein	1	5,9
KER07271.1	hypothetical protein SK271_1337	1	1,5
KER07317.1	hypothetical protein SK271_1383	1	7,2
KER07346.1	1,4-beta-N-acetylmuramidase	1	2,6
KER07408.1	serine/threonine protein phosphatase Stp1	1	0,4
KER07414.1	ribonuclease Y	1	2,4

KER07417.1	hypothetical protein SK271_1483	1	8,5
KER07443.1	malonyl CoA-acyl carrier protein transacylase	1	5,5
KER07448.1	enoyl-CoA hydratase/isomerase family protein	1	5,7
KER07475.1	cell cycle protein gpsB	1	24,8
KER07485.1	hypothetical protein SK271_1551	1	3,6
KER07496.1	polysaccharide export, MPA1 family protein	1	7,0
KER07499.1	bacterial extracellular solute-binding s, 5 Middle family protein	1	2,3
KER07503.1	putative ATP-dependent Clp protease ATP-binding subunit	1	2,3
KER07574.1	hypothetical protein SK271_1640	1	2,2
KER07586.1	protein RecA	1	3,9
KER07651.1	acetyltransferase family protein	1	8,3
KER07782.1	translation elongation factor Ts	1	4,6
KER07853.1	HAD hydrolase, IIB family protein	1	6,3
KER07891.1	serine hydroxymethyltransferase family protein	1	3,1
KER07904.1	large conductance mechanosensitive channel protein	1	17,5
KER07917.1	riboflavin biosynthesis protein RibF	1	4,3
KER07988.1	3-isopropylmalate dehydrogenase	1	3,8
KER07999.1	phosphotransferase enzyme family protein	1	3,8
KER08004.1	licD family protein	1	6,3
KER08086.1	bacterial capsule synthesis PGA_cap family protein	1	3,4
KER08103.1	NADPH-dependent FMN reductase family protein	1	4,5
KER08108.1	glutathione-dependent formaldehyde-activating enzyme family protein	1	21,9
KER08116.1	PTS system, glucose subfamily, IIA component domain protein	1	2,5
KER08154.1	hydroxyethylthiazole kinase	1	7,3
KER08164.1	POTRA domain, FtsQ-type family protein	1	3,1
KER08324.1	3-deoxy-7-phosphoheptulonate synthase	1	3,2
KER08344.1	penicillin-binding protein 2B	1	2,0
KER08406.1	30S ribosomal protein S15	1	10,4
KER08412.1	pfkB carbohydrate kinase family protein	1	4,3
KER08477.1	3-dehydroquinate dehydratase	1	5,5
KER08480.1	chorismate synthase	1	3,4
KER08482.1	hypothetical protein SK271_0525	1	9,8
KER08526.1	efflux transporter, RND family, MFP subunit	1	5,0
KER08528.1	ftsX-like permease family protein	1	5,1
KER08583.1	bacterial extracellular solute-binding s, 5 Middle family protein	1	2,3
KER08594.1	D-alanyl-D-alanine carboxypeptidase	1	3,9
KER08682.1	arginine repressor	1	9,1
KER08699.1	lactose phosphotransferase system repressor	1	3,5
KER08781.1	catabolite control protein A	1	4,2
KER08783.1	HAD hydrolase, IIB family protein	1	2,5
KER08788.1	bacterial extracellular solute-binding s, 5 Middle family protein	1	2,3
KER08806.1	aspartate--ammonia ligase	1	3,0
KER08809.1	lon protease (S16) C-terminal proteolytic domain protein	1	5,8

KER08841.1	UDP-glucose 4-epimerase	1	3,9
KER08870.1	thioredoxin family protein	1	2,6
KER08890.1	translation initiation factor IF-2	1	2,0
KER08925.1	lysM domain protein	1	3,3
