# Supplementary material for: Proteotyping bacteria: Characterization, differentiation and identification of pneumococcus and other species within the Mitis Group of the genus Streptococcus by tandem mass spectrometry proteomics
Source: PLoS One. 2018 Dec 10;13(12):e0208804. doi: 10.1371/journal.pone.0208804 (PMC6287849; doi:10.1371/journal.pone.0208804)
Supplement: S10 Table — (PDF) [file pone.0208804.s010.pdf]

**S10 Table.**

**List of proteins identified by species-unique peptides in analysis of *S. pseudopneumoniae* CCUG 49455T**

| Accession number | Description | Nº peptides | Coverage |
|---|---|---|---|
| ORC37164.1 | cell wall-binding protein, partial | 43 | 47,9 |
| ORC38886.1 | choline-binding protein | 21 | 54,3 |
| ORC39808.1 | hypothetical protein B4W83_06435, partial | 18 | 35,7 |
| ORC40644.1 | hypothetical protein B4W83_04385, partial | 16 | 20,4 |
| ORC39803.1 | secretion protein | 15 | 36,6 |
| ORC40058.1 | hypothetical protein B4W83_05940, partial | 14 | 27,5 |
| ORC41371.1 | peptidase M26 | 14 | 8,5 |
| ORC39560.1 | hypothetical protein B4W83_07325, partial | 8 | 38,7 |
| ORC42535.1 | peptidase, partial | 7 | 7,7 |
| ORC36032.1 | peptidase, partial | 6 | 11,6 |
| ORC36825.1 | YSIRK signal domain/LPXTG anchor domain surface protein | 6 | 6,4 |
| ORC42021.1 | hypothetical protein B4W83_01405 | 6 | 16,9 |
| ORC35201.1 | hypothetical protein B4W83_11775, partial | 5 | 10,8 |
| ORC36031.1 | hypothetical protein B4W83_11525 | 3 | 1,3 |
| ORC36014.1 | alpha-glycerophosphate oxidase | 3 | 6,9 |
| ORC39780.1 | hypothetical protein B4W83_06530 | 3 | 5,1 |
| ORC39539.1 | AAA family ATPase | 3 | 8,3 |
| ORC39626.1 | 30S ribosomal protein S1 | 3 | 4,0 |
| ORC38895.1 | choline-binding protein C | 2 | 6,9 |
| ORC40501.1 | transposase | 2 | 8,1 |
| ORC41944.1 | pullulanase | 2 | 3,3 |
| ORC34791.1 | growth inhibitor PemK | 2 | 9,8 |
| ORC39603.1 | hemolysin | 2 | 2,9 |
| ORC38998.1 | sugar ABC transporter substrate-binding protein | 2 | 6,1 |
| ORC40049.1 | catabolite control protein A | 2 | 7,4 |
| ORC42007.1 | 50S ribosomal protein L3 | 2 | 8,7 |
| ORC40377.1 | cell division protein SepF | 2 | 16,2 |
| ORC41209.1 | 6-phospho-beta-glucosidase | 2 | 6,1 |
| ORC41496.1 | site-specific DNA-methyltransferase | 1 | 2,6 |
| ORC39414.1 | protein jag | 1 | 4,0 |
| ORC39417.1 | acetate kinase | 1 | 3,8 |
| ORC37105.1 | tagatose 1,6-diphosphate aldolase | 1 | 4,3 |
| ORC36026.1 | SPFH domain-containing protein | 1 | 3,0 |
| ORC36181.1 | DNA-directed RNA polymerase subunit beta' | 1 | 0,8 |
| ORC37108.1 | galactose-6-phosphate isomerase subunit LacA | 1 | 7,8 |
| ORC39776.1 | hypothetical protein B4W83_06490 | 1 | 8,5 |
| ORC36179.1 | DNA-directed RNA polymerase subunit beta | 1 | 1,6 |
| ORC40508.1 | bacteriocin ABC transporter ATP-binding protein | 1 | 2,4 |
| ORC41249.1 | foldase PrsA | 1 | 3,5 |
| ORC36797.1 | alanine--tRNA ligase | 1 | 1,5 |
| ORC41690.1 | phosphoglucosamine mutase | 1 | 3,3 |
| ORC42020.1 | dehydrogenase | 1 | 4,3 |
| ORC39960.1 | ribonuclease Y | 1 | 1,9 |
| ORC39593.1 | ATP-dependent Clp protease ATP-binding subunit | 1 | 1,6 |
| ORC41645.1 | ATP synthase F0 subunit B | 1 | 7,3 |
| ORC41943.1 | thiol-activated cytolysin | 1 | 2,3 |

| | | | |
|---|---|---|---|
| ORC35202.1 | hypothetical protein B4W83_11780 | 1 | 5,3 |
| ORC41216.1 | serine hydroxymethyltransferase | 1 | 3,1 |
| ORC40884.1 | sucrose-6-phosphate hydrolase | 1 | 3,1 |
| ORC41631.1 | phosphoglucomutase | 1 | 3,0 |
| ORC41992.1 | 50S ribosomal protein L6 | 1 | 7,9 |
| ORC41959.1 | proline--tRNA ligase | 1 | 2,8 |
| ORC42553.1 | pyruvate oxidase | 1 | 2,4 |
| ORC41665.1 | manganese-dependent inorganic pyrophosphatase | 1 | 6,4 |
| ORC34580.1 | restriction endonuclease, partial | 1 | 6,3 |
| ORC38991.1 | tyrosine--tRNA ligase | 1 | 3,3 |
| ORC37089.1 | dihydroxyacetone kinase subunit DhaK | 1 | 4,3 |
| ORC39639.1 | PTS fructose transporter subunit IIC | 1 | 2,9 |
| ORC37163.1 | hypothetical protein B4W83_10270, partial | 1 | 7,9 |
| ORC39488.1 | FRG domain-containing protein | 1 | 4,0 |
| ORC42538.1 | L-lactate oxidase | 1 | 4,5 |
| ORC42022.1 | hypothetical protein B4W83_01410 | 1 | 5,7 |
| ORC41389.1 | lactococcin 972 family bacteriocin | 1 | 12,2 |
| ORC42502.1 | hypothetical protein B4W83_00565 | 1 | 9,8 |
| ORC40053.1 | universal stress protein UspA | 1 | 15,3 |
| ORC41659.1 | peptide ABC transporter ATP-binding protein | 1 | 2,3 |
| ORC35836.1 | IS1634 family transposase | 1 | 2,1 |
| ORC38937.1 | GntR family transcriptional regulator | 1 | 5,2 |
| ORC41928.1 | aminopeptidase | 1 | 2,3 |
| ORC36817.1 | phosphate-binding protein | 1 | 4,1 |
| ORC40999.1 | MarR family transcriptional regulator | 1 | 10,4 |
| ORC42517.1 | multidrug ABC transporter ATP-binding protein | 1 | 2,4 |
| ORC41244.1 | bifunctional N-acetylglucosamine-1-phosphate uridyltransferase/glucosamine-1-phosphate acetyltransferase | 1 | 2,6 |
| ORC41480.1 | propanediol utilization protein | 1 | 9,9 |
| ORC40670.1 | DNA-binding response regulator | 1 | 6,0 |
| ORC38577.1 | RNA-binding transcriptional accessory protein | 1 | 3,0 |
| ORC38011.1 | phosphorylcholine transferase LicD | 1 | 6,3 |
| ORC41394.1 | cell division protein DivIB | 1 | 4,7 |
| ORC40025.1 | GTP-sensing pleiotropic transcriptional regulator CodY | 1 | 6,1 |
| ORC37463.1 | hypothetical protein B4W83_10170 | 1 | 3,1 |

ç
