# Supplementary material for: Proteotyping bacteria: Characterization, differentiation and identification of pneumococcus and other species within the Mitis Group of the genus Streptococcus by tandem mass spectrometry proteomics
Source: PLoS One. 2018 Dec 10;13(12):e0208804. doi: 10.1371/journal.pone.0208804 (PMC6287849; doi:10.1371/journal.pone.0208804)
Supplement: S11 Table — (PDF) [file pone.0208804.s011.pdf]

S11 Table.

List of proteins identified by species-unique peptides in analysis of *S. pseudopneumoniae* CCUG 62647

Accession number	Description	Nº peptides	Coverage
OOR79191.1	cell wall-binding protein, partial	27	35,3
OOR84059.1	choline-binding protein	20	42,3
OOR85723.1	hypothetical protein B0177_00115, partial	16	34,3
OOR84336.1	peptidase M26	13	9,3
OOR81496.1	hypothetical protein B0177_08140, partial	12	44,7
OOR80376.1	peptidase	11	9,6
OOR81490.1	secretion protein	10	29,2
OOR81829.1	hypothetical protein B0177_07365	5	8,8
OOR81958.1	hypothetical protein B0177_06775	5	15,7
OOR83686.1	hypothetical protein B0177_02640, partial	5	7,3
OOR79078.1	hemolysin	4	6,9
OOR79405.1	alpha-glycerophosphate oxidase	4	7,1
OOR81957.1	hypothetical protein B0177_06770	4	14,7
OOR82036.1	pullulanase	4	7,0
OOR83042.1	catabolite control protein A	4	10,7
OOR81959.1	dehydrogenase	3	13,9
OOR82037.1	thiol-activated cytolysin	3	4,5
OOR81825.1	hypothetical protein B0177_07325	2	24,6
OOR81973.1	50S ribosomal protein L3	2	8,7
OOR82171.1	pyruvate oxidase	2	4,7
OOR83544.1	peptide ABC transporter ATP-binding protein	2	5,1
OOR84308.1	lysine--tRNA ligase	2	3,2
OOR85073.1	hypothetical protein B0177_00560	2	18,2
OOR85206.1	foldase PrsA	2	6,1
OOR85742.1	AAA family ATPase	2	7,6
OOR78195.1	hypothetical protein B0177_11640, partial	1	16,3
OOR78926.1	DNA-directed RNA polymerase subunit beta	1	1,6
OOR79025.1	choline-binding protein	1	3,2
OOR79042.1	1-phosphofructokinase	1	4,6
OOR79055.1	30S ribosomal protein S1	1	2,5
OOR79129.1	hypothetical protein B0177_10505	1	3,1
OOR79418.1	beta-galactosidase	1	1,0
OOR79423.1	hypothetical protein B0177_10235	1	0,6
OOR79424.1	peptidase, partial	1	3,0
OOR79501.1	ABC transporter	1	5,2
OOR79705.1	potassium-transporting ATPase subunit C	1	8,3
OOR79716.1	phosphoglucomutase	1	3,0
OOR79727.1	ATP synthase subunit alpha	1	3,8
OOR79729.1	ATP synthase F0 subunit B	1	7,3
OOR80498.1	FRG domain-containing protein	1	4,0
OOR80503.1	Fic/DOC family protein	1	3,8
OOR80894.1	GMP synthetase	1	1,9
OOR81236.1	hypothetical protein B0177_08175	1	10,8
OOR81256.1	phosphate-binding protein	1	4,1
OOR81715.1	ribonucleoside-diphosphate reductase subunit alpha	1	1,9
OOR81771.1	CHAP domain-containing protein	1	3,1
OOR81835.1	phosphoglycolate phosphatase	1	4,8

OOR81924.1	ABC transporter permease	1	10,7
OOR82115.1	efflux transporter periplasmic adaptor subunit	1	4,3
OOR82186.1	L-lactate oxidase	1	4,5
OOR82191.1	mannose-6-phosphate isomerase, class I	1	6,1
OOR82226.1	serine/threonine protein kinase	1	2,4
OOR82232.1	sucrose-6-phosphate hydrolase	1	3,1
OOR82287.1	ATP-dependent Clp protease ATP-binding subunit	1	1,6
OOR82513.1	tRNA (adenine-N(1))-methyltransferase	1	7,6
OOR82545.1	cell division protein SepF	1	15,6
OOR82564.1	sugar ABC transporter substrate-binding protein	1	2,3
OOR82565.1	PTS glucose transporter subunit IIBC	1	3,4
OOR82583.1	preprotein translocase subunit SecA	1	1,3
OOR82682.1	helicase BlpT	1	10,3
OOR82689.1	molecular chaperone DnaK	1	1,8
OOR82694.1	restriction endonuclease subunit R	1	1,4
OOR82770.1	hypothetical protein B0177_04075	1	8,0
OOR83096.1	ribonuclease Y	1	1,9
OOR83547.1	CsbD family protein	1	21,9
OOR83598.1	transcriptional repressor CodY	1	6,1
OOR83692.1	arginine repressor	1	11,9
OOR83714.1	DNA-binding response regulator	1	6,0
OOR83761.1	DNA protecting protein DprA	1	5,6
OOR84337.1	TIGR01440 family protein	1	7,7
OOR84807.1	sugar ABC transporter substrate-binding protein	1	3,3
OOR84815.1	tyrosine--tRNA ligase	1	3,3
OOR84853.1	acetate kinase	1	3,8
OOR84856.1	protein jag	1	4,0
OOR84874.1	permease	1	4,6
OOR85049.1	starch synthase	1	3,6
OOR85121.1	hypothetical protein B0177_00820	1	31,1
OOR85143.1	hypothetical protein B0177_00940	1	4,2
OOR85173.1	serine hydroxymethyltransferase	1	3,1
