# Supplementary material for: Proteotyping bacteria: Characterization, differentiation and identification of pneumococcus and other species within the Mitis Group of the genus Streptococcus by tandem mass spectrometry proteomics
Source: PLoS One. 2018 Dec 10;13(12):e0208804. doi: 10.1371/journal.pone.0208804 (PMC6287849; doi:10.1371/journal.pone.0208804)
Supplement: S12 Table — (PDF) [file pone.0208804.s012.pdf]

S12 Table.
List of proteins identified by species-unique peptides in analysis of *S. pseudopneumoniae* CCUG 63747

Accession number	Description	Nº peptides	Coverage
OOR81280.1	hypothetical protein B0178_10245, partial	30	21,3
OOR78974.1	hypothetical protein B0178_11945	22	29,7
OOR83641.1	choline-binding protein	17	42,3
OOR86110.1	hypothetical protein B0178_04610	15	12,2
OOR86154.1	peptidase M26	15	8,5
OOR85830.1	secretion protein	12	27,6
OOR83161.1	hypothetical protein B0178_08355, partial	9	74,8
OOR85824.1	hypothetical protein B0178_05325, partial	8	50,7
OOR85877.1	hypothetical protein B0178_05310, partial	6	19,9
OOR86918.1	hypothetical protein B0178_00525	5	15,9
OOR80748.1	alpha-glycerophosphate oxidase	4	7,1
OOR85899.1	hypothetical protein B0178_05190	4	8,8
OOR86243.1	hypothetical protein B0178_03410, partial	4	7,4
OOR81272.1	catabolite control protein A	3	7,7
OOR85903.1	hypothetical protein B0178_05230	3	33,8
OOR86338.1	YSIRK signal domain/LPXTG anchor domain surface protein	3	3,3
OOR86838.1	thiol-activated cytolysin	3	4,5
OOR86917.1	dehydrogenase	3	19,6
OOR86919.1	hypothetical protein B0178_00530	3	8,4
OOR82457.1	peptide ABC transporter ATP-binding protein	2	5,1
OOR83139.1	AAA family ATPase	2	7,2
OOR83160.1	hypothetical protein B0178_08350, partial	2	11,3
OOR83642.1	choline-binding protein C	2	6,9
OOR85011.1	CHAP domain-containing protein	2	6,4
OOR86079.1	cell division protein SepF	2	16,2
OOR86139.1	beta-galactosidase	2	1,3
OOR86379.1	hypothetical protein B0178_02025	2	18,2
OOR86475.1	6-phospho-beta-glucosidase	2	6,1
OOR86516.1	foldase PrsA	2	6,1
OOR86760.1	pyruvate oxidase	2	4,7
OOR86839.1	pullulanase	2	3,3
OOR86903.1	50S ribosomal protein L3	2	8,7
OOR79007.1	peptide ABC transporter ATP-binding protein	1	2,6
OOR79186.1	ATP-dependent Clp protease ATP-binding subunit	1	1,6
OOR80082.1	DNA-directed RNA polymerase subunit beta	1	1,6
OOR80390.1	GMP synthetase	1	1,9
OOR80762.1	hypothetical protein B0178_10835, partial	1	18,7
OOR81511.1	ABC transporter	1	5,2
OOR81799.1	serine/threonine protein kinase	1	2,4
OOR81810.1	sodium ABC transporter permease	1	4,0
OOR81872.1	tyrosine--tRNA ligase	1	3,3
OOR82428.1	phosphoglucomutase	1	3,0
OOR82440.1	ATP synthase subunit alpha	1	3,8
OOR82442.1	ATP synthase F0 subunit B	1	7,3
OOR82460.1	CsbD family protein	1	21,9
OOR82463.1	manganese-dependent inorganic pyrophosphatase	1	6,4
OOR82476.1	NAD(P)H-dependent oxidoreductase	1	7,1

OOR83788.1	acetate kinase	1	3,8
OOR83791.1	protein jag	1	4,0
OOR83807.1	permease	1	4,6
OOR84437.1	exodeoxyribonuclease VII large subunit	1	3,1
OOR84745.1	Fic/DOC family protein	1	3,8
OOR84949.1	dihydroxyacetone kinase subunit DhaK	1	4,3
OOR84968.1	ribonuclease Y	1	1,9
OOR85693.1	haloacid dehalogenase	1	5,6
OOR85797.1	MarR family transcriptional regulator	1	10,4
OOR85930.1	restriction endonuclease subunit R	1	1,4
OOR86039.1	preprotein translocase subunit SecA	1	1,3
OOR86060.1	sugar ABC transporter substrate-binding protein	1	2,3
OOR86135.1	transcriptional repressor CodY	1	6,1
OOR86153.1	TIGR01440 family protein	1	7,7
OOR86182.1	lysine--tRNA ligase	1	3,2
OOR86187.1	potassium-transporting ATPase subunit C	1	8,3
OOR86230.1	glycine--tRNA ligase subunit beta	1	2,4
OOR86330.1	phosphate-binding protein	1	4,1
OOR86426.1	hypothetical protein B0178_02270	1	4,2
OOR86428.1	hypothetical protein B0178_02280, partial	1	31,5
OOR86482.1	serine hydroxymethyltransferase	1	3,1
OOR86888.1	50S ribosomal protein L6	1	10,7
