# Supplementary material for: Proteotyping bacteria: Characterization, differentiation and identification of pneumococcus and other species within the Mitis Group of the genus Streptococcus by tandem mass spectrometry proteomics
Source: PLoS One. 2018 Dec 10;13(12):e0208804. doi: 10.1371/journal.pone.0208804 (PMC6287849; doi:10.1371/journal.pone.0208804)
Supplement: S13 Table — (PDF) [file pone.0208804.s013.pdf]

S13 Table. Number of peptide matches for S2 Figure, showing the species assignments using the Initial vs the Curated Databases

PEPTIDE MATCHES DATABASE CURATED

	S. pneumoniae			*S. pseudopneumoniae*			*S. mitis*			*S. pyogenes*		
	CCUG 28588T	CCUG 7206	CCUG 35180	CCUG 49455T	CCUG 63747	CCUG 62647	CCUG 31611T	CCUG 63687	CCUG 69183	CCUG 4207T	CCUG 25570	CCUG 47803
S. pneumoniae	220	175	214									
S. pseudopneumoniae				433	245	250						
S. mitis						7	272	287	500			
S. pyogenes										314	351	418
S. suis	7								6			

PEPTIDE MATCHES DATABASE INITIAL

	S. pneumoniae			*S. pseudopneumoniae*			*S. mitis*			*S. pyogenes*		
	CCUG 28588T	CCUG 7206	CCUG 35180	CCUG 49455T	CCUG 63747	CCUG 62647	CCUG 31611T	CCUG 63687	CCUG 69183	CCUG 4207T	CCUG 25570	CCUG 47803
S. pneumoniae	347	286	380	74	26	30	34	21	35			
S. pseudopneumoniae				230	183	189	21	17	37			
S. mitis				18		11	272	144	134			
S. pyogenes										319	360	427
S. suis	7								6			
S. oralis				7	5		16	12				
S. parasanguinis					18							
B. proteoclasticus							34	7	15			
